# Temporal Trajectories of the Tau Aggregate Interactome Reveal Stage‐Specific Vulnerabilities in Alzheimer's Disease

**DOI:** 10.1002/advs.77822

**Published:** 2026-09-16

**Authors:** Dorothea Böken, Paula Beltran Lobo, Yunzhao Wu, Lyla A. Rowe, Emre Fertan, Cara L. Croft, Maria Jimenez‐Sanchez, Dezerae Cox, David Klenerman

**Affiliations:** ^1^ Yusuf Hamied Department of Chemistry University of Cambridge Cambridge UK; ^2^ UK Dementia Research Institute University of Cambridge Cambridge UK; ^3^ Department of Basic and Clinical Neuroscience, Maurice Wohl Clinical Neuroscience Institute King's College London London UK; ^4^ Centre For Neuroscience, Surgery and Trauma, The Blizard Institute Queen Mary University of London London UK; ^5^ Department of Clinical Neurosciences University of Cambridge Cambridge UK; ^6^ Molecular Horizons, School of Sciences University of Wollongong Wollongong New South Wales Australia

**Keywords:** Alzheimer's disease, interactome, proteomics, single‐molecule, Tau

## Abstract

Tau aggregation is a central pathological feature of Alzheimer's disease, yet how different forms of tau—ranging from monomers to small soluble aggregates and mature fibrils—interact with the cellular environment remains poorly understood. Here, we combine immunoaffinity proteomics with single‐molecule techniques and super‐resolution microscopy to systematically map the tau interactome across defined aggregation states, spanning monomeric tau, nanoscopic soluble aggregates, and fibrillar species. Using post‐mortem Alzheimer's disease brain tissue, we identify distinct functional modules associated with different aggregation states: while proteostasis factors and immune‐related proteins preferentially associate with nanoscopic aggregates (oligomers), cytoskeletal, metabolic, and RNA‐binding proteins are enriched for mature fibrillar tau. Single‐molecule microscopy directly confirms this conformation‐dependent recruitment for key interactors including Hsp70‐2, ENO1, hnRNPA1, APP, EAAT4, and ubiquitin. A primary‐neuron system with accelerated tau aggregation is used to model these findings in a controlled system, showing striking similarities to the brain samples. Finally, pseudotime analysis reconstructs a progressive remodelling of the tau interactome across disease progression, revealing stage‐specific pathway vulnerabilities. Together, these results establish a temporally resolved framework for tau pathology shaped by protein interactions and identify potential therapeutic intervention points for investigation across stages of disease.

## Graphical abstract

Tau populations with different aggregation states associate with distinct sets of proteins in the Alzheimer's disease brain. Proteomic discovery combined with single‐molecule analysis in neurons reveals dynamic associations involving proteostasis, metabolism and RNA biology. These state‐associated interactomes highlight changing molecular vulnerabilities during tau aggregation.

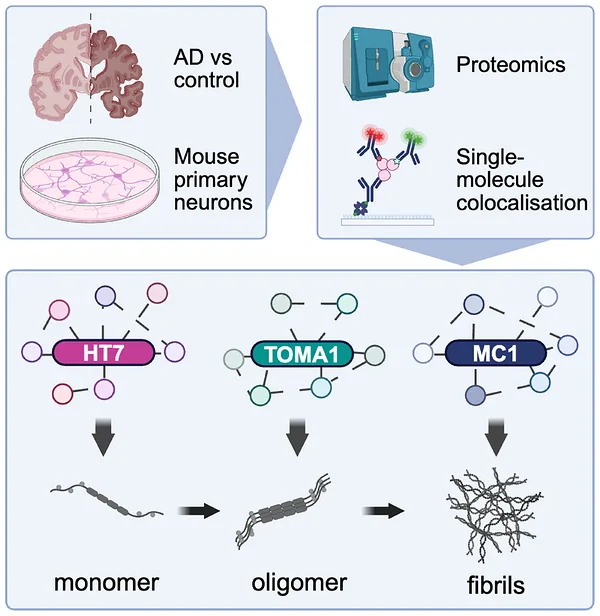

## 1 Introduction

Tauopathies, including Alzheimer's disease (AD), are characterized by the pathological aggregation of the microtubule‐associated protein tau [1]. Under physiological conditions, tau is monomeric, largely unaggregated, and dynamically regulated through reversible phosphorylation. In disease, tau becomes hyperphosphorylated, detaches from microtubules, and assembles into soluble oligomers and ultimately into insoluble fibrils. Particularly the smaller, earlier aggregate species are implicated in synaptic dysfunction [2], proteostasis failure [3], and neuronal toxicity [4], yet the precise molecular mechanism(s) linking tau aggregation to neurodegeneration remain inadequately understood.

A critical but underexplored aspect of tau pathology is how its interactome changes during different stages of aggregation, since these changing protein associations likely mediate tau's cellular toxicity. Tau interacts with diverse cellular partners, including chaperones, cytoskeletal regulators, metabolic enzymes, and degradation machinery [5]. These associations would be expected to evolve as tau progresses from monomers to small aggregates (oligomeric) and, ultimately, mature fibrils [6, 7]. While neurofibrillary tangles (NFTs) are a hallmark of AD, increasing evidence points to smaller tau aggregates as the most neurotoxic form [8, 9]. Defining which proteins associate with tau at specific aggregation states, and how post‐translational modifications (PTMs) shape these interactions, is therefore essential for understanding tau‐mediated toxicity.

Resolving these interactions has been technically challenging. Traditional approaches, including proteomics and conventional immunoprecipitation, have provided valuable insight into tau‐associated proteins and implicated key pathways in disease [6, 10, 11]. However, these methods typically analyze bulk tau preparations and thus are limited in their ability to distinguish interactions specific to distinct conformational or aggregation states. The small, early tau aggregate species are particularly difficult to capture as they are low in abundance, heterogeneous in structure, and often masked by more abundant forms [12]. In contrast, single‐molecule techniques enable direct visualization of individual aggregates and their associated proteins, allowing us to track tau interactions across aggregation states [13]. However, single‐molecule methods, while offering molecular resolution, are limited in throughput and typically probe only a subset of proteins, making it difficult to capture the full complexity of tau's interactome [12]. Here, we combine aggregate state‐specific proteomic profiling with single‐molecule imaging to map the tau interactome from monomers to fibrils, capturing both global trends and molecular‐scale insights that define stage‐specific vulnerabilities in tau pathology.

Here, to systematically investigate how tau's interactome changes in AD, we first performed exploratory proteomic profiling of tau‐associated proteins from post‐mortem AD and control brain tissue using a panel of antibodies targeting distinct conformational and phosphorylation states [14, 15, 16]. Based on differential enrichment patterns identified in this dataset, we selected a subset of candidate interactors for orthogonal validation and further characterization using single‐molecule imaging in human brain tissue and in a longitudinal primary neuron model expressing human wild‐type or aggregation‐prone mutant human tau (P301L/S320F) [14]. Finally, by aligning proteomic signatures across tau conformers, we inferred a pseudotemporal trajectory of tau aggregation. This revealed a staged remodeling of protein interactions, from early association of proteostasis and immune regulators to late‐state associations with metabolic, cytoskeletal, and synaptic components. This integrated approach establishes a temporal framework for tau aggregation and its molecular context, highlighting potential vulnerabilities that may inform stage‐specific therapeutic interventions.

## 2 Results

### 2.1 Proteomic Profiling of Tau Interactors across Aggregation States in AD

To characterize the interactome associated with different physicochemical properties of tau, we performed immunoprecipitations using five tau‐targeting antibodies: HT7 (pan tau), TOMA1 [15] (oligomeric tau), MC1 [16] (fibrillar tau), and two phospho‐tau antibodies, AT8 [17] (an established pathological tau marker recognizing phosphorylated S202/phosphorylated T205) and T181 (phosphorylated T181). To assess the extent to which HT7, TOMA1 and MC1 distinguish between tau aggregate states, we compared their reactivity against monomeric tau, small tau aggregates, heparin‐assembled tau aggregates and fibrils isolated from Alzheimer's disease brain tissue, confirming their expected differential reactivity across these species (Figure S1). Target tau complexes were immunoprecipitated from post‐mortem frontal cortex (BA6/8) of four individuals with Alzheimer's disease (AD, Braak stage VI) and five age‐matched controls (HC, Braak stage 0) (Table 1). The tau‐associated proteins were then identified and quantified by mass spectrometry.

**TABLE 1:** Patient information brain homogenate.

Patient	Sex	Age [years]	Braak Stage	Region	BBN
**AD1**	Female	72	VI	BA6/8	BBN001.36924
**AD2**	Male	75	VI	BA6/8	BBN001.37400
**AD3**	Female	85	VI	BA6/8	BBN_25739
**AD4**	Male	75	VI	BA6/8	BBN001.36839
**AD5**	Male	75	VI	BA6/8	BBN001.36689
**HC1**	Female	71	0	BA6/8	BBN001.29882
**HC2**	Male	72	0	BA6/8	BBN001.30178
**HC3**	Female	73	0	BA6/8	BBN001.35138
**HC4**	Male	71	0	BA6/8	BBN001.30916
**HC5**	Male	82	0	BA6/8	BBN001.35549

**FIGURE 1:**
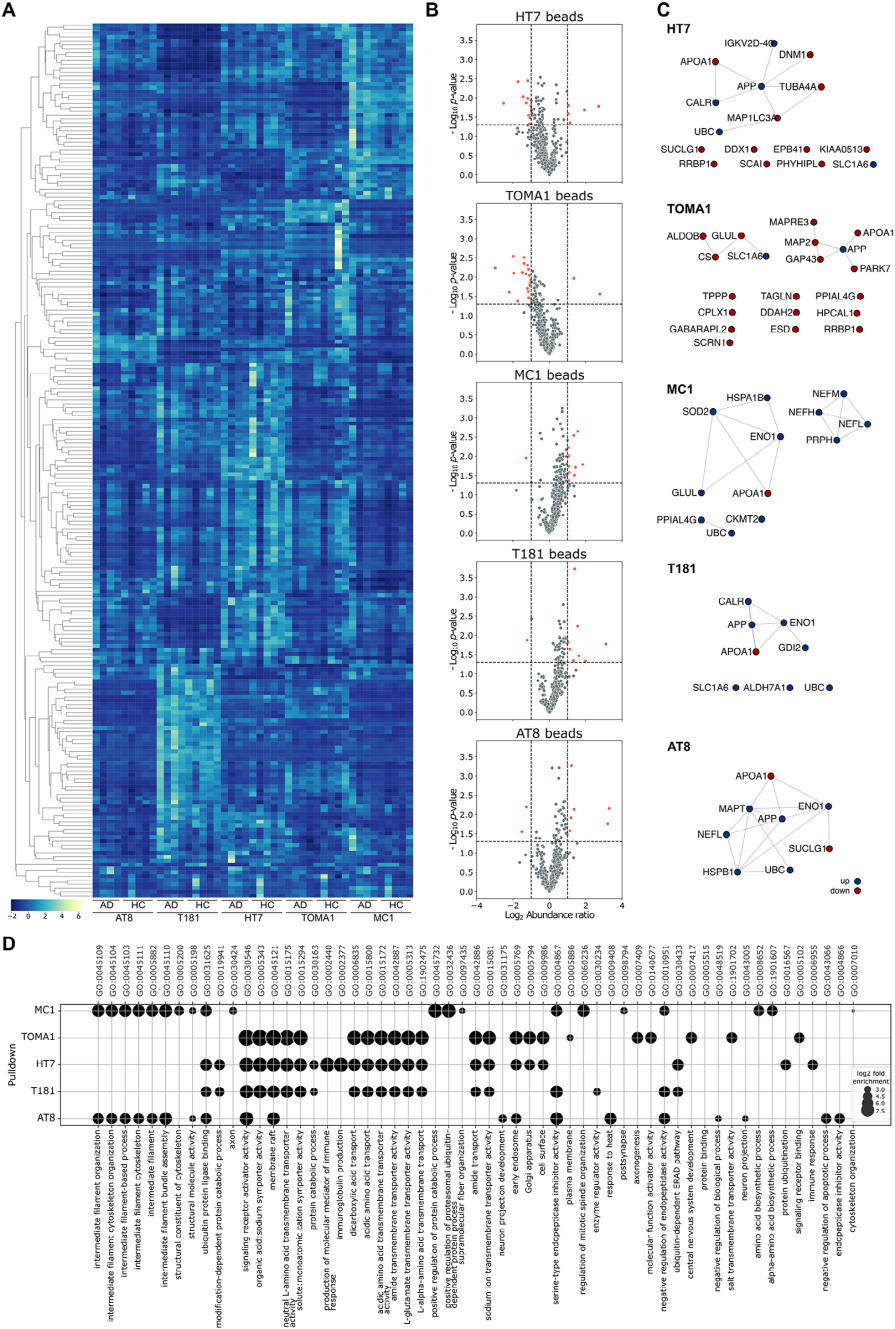
State‐specific profiling of the tau interactome in Alzheimer's disease using immunoprecipitation proteomics. (A) Heatmap showing hierarchical clustering of tau interactors from immunoprecipitations performed on post‐mortem brain tissue from AD and age‐matched control donors (*n* = 5 HC, 4 AD after QC filtering). Tau was immunoprecipitated using five antibodies targeting distinct conformational and phosphorylation states (HT7, TOMA1, MC1, AT8, T181), followed by mass spectrometry. (B) Volcano plots showing differential enrichment of tau interactors between AD and HC for each antibody. Red points indicate candidate proteins meeting the exploratory thresholds of |log_2_ fold change| > 1, *p* < 0.05. (C) STRING network visualisations of the candidate interactors. (D) Gene Ontology enrichment analysis of candidate interactors for each tau species. Bubble size represents the number of genes enriched per term.

In total, 1193 unique proteins were identified across the five antibody specific tau interactomes. Unsupervised hierarchical clustering of these proteins (Figure 1A) was dominated by the target antibody type, confirming that tau conformation and post‐translational state are more dominant drivers of variations in the tau interactome than AD itself. Therefore, to identify disease‐associated differences in the interactome of each target tau, we examined the fold difference in the amount of each interactor (log_2_ AD/HC abundance; Figure 1B). To nominate candidate disease‐associated differences, we applied exploratory thresholds of |log_2_ fold change| > 1 and *p* < 0.05. We found that 46 proteins met these criteria. These thresholds were used to prioritize candidates for further investigation rather than to define a confirmatory set of differentially associated proteins. The limited number of statistically significant hits likely reflects the considerable biological heterogeneity inherent to post‐mortem brain which can obscure consistent interactome shifts. To assess inter‐individual variability, we examined donor‐level log_2_ AD/mean HC abundance ratios for the top interactors within each antibody‐defined interactome. These changes were broadly consistent in direction and magnitude across the donors, indicating that they were not driven by a single outlier individual (Figure S2). Other proteins, such as hnRNPA1, were not quantified in the bound tau interactome but were differentially abundant in the tau‐depleted supernatants suggesting these may be transient interactors (Figure S3A–F).

Distinct interactomes emerged for each tau species. For example, HT7 pulldowns were enriched in proteostasis‐related proteins (e.g., CALR, MAP1LC3A), while the MC1 pulldown featured cytoskeletal elements (e.g., NEFL, PRPH) and chaperones (e.g., HSPA1B). These AD‐related modulations were largely target‐specific; only 11 interactors were significantly enriched in more than one antibody interactome. Interestingly, the direction of change also varied by tau species: for HT7, MC1, and the phospho‐tau antibodies, most significant interactors were upregulated in AD. In contrast, for TOMA1 21 out of 23 differentially enriched proteins were decreased in AD. This suggests an aggregation state‐specific loss of protein interactions at the oligomeric tau state, potentially reflecting early pathological disruption, and gain of protein interaction at the fibrillar and phosphorylated tau. Despite the relatively modest number of identified candidate interactors, those altered in AD consistently formed highly connected networks (Figure 1C; STRINGdb). For AT8, TOMA1, and MC1, the observed protein–protein interactions were significantly more connected than expected by chance (STRING PPI enrichment p < 0.05), while T181 and HT7 showed a strong trend (T181: p  =  0.0582, HT7: p  =  0.0935). The overall connectivity across tau species supports the functional coherence of these AD‐altered interactomes.

To understand the functional implications of interactome differences in AD, we performed gene ontology (GO) enrichment analysis on the identified candidate interactors (Figure 1D). Each antibody‐specific tau interactome is associated with the modulation of distinct biological processes in AD; primarily monomeric tau (HT7) was associated with proteolytic quality control and immune activation pathways, suggesting early stress responses, while oligomeric tau (TOMA1) showed dysregulation of synaptic processes, metabolic regulation, and neuronal morphogenesis, indicative of disrupted cellular homeostasis. In contrast, fibrillar tau (MC1) was enriched for cytoskeletal reorganization and post‐synaptic structure proteins, reflecting the position of fibrillar tau aggregates as end‐stage pathologies. Notably, the enrichment revealed two clear antibody‐related groupings. MC1 and AT8, which both recognize more conformationally altered/late stage aggregated forms of tau, showed highly similar functional enrichment profiles. Conversely, HT7, TOMA1 and T181, which recognize earlier, soluble states, showed greater similarity to one another. This highlights that antibodies recognizing similar tau species yield correspondingly similar enrichment profiles.

### 2.2 Progressive Tau Aggregation and Phosphorylation in Primary Neurons Expressing Aggregation‐Prone Human Tau

**FIGURE 2:**
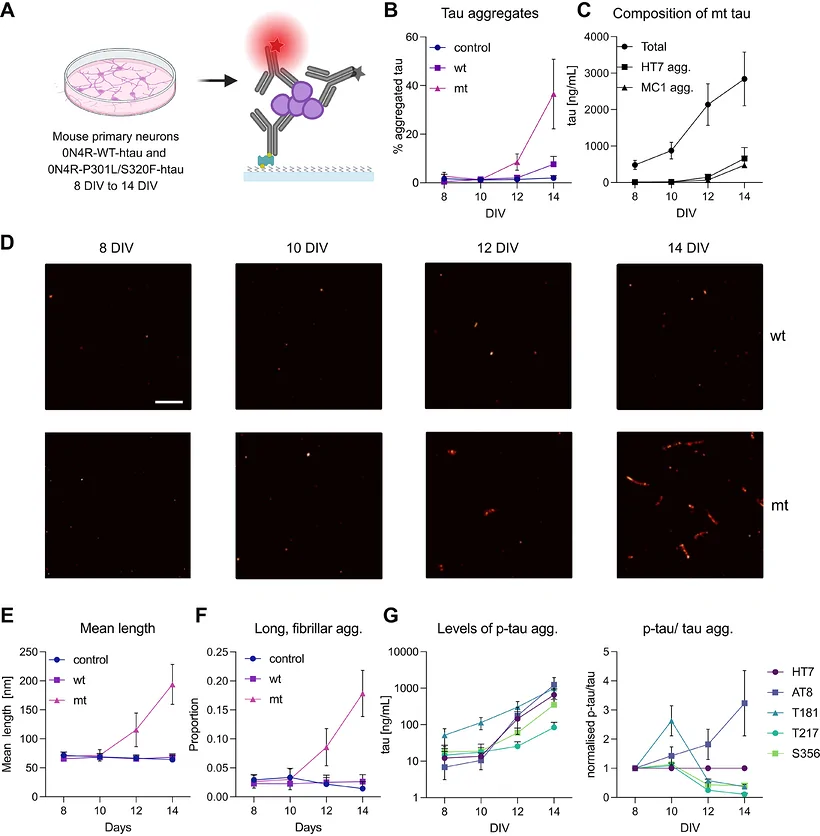
Tau aggregation dynamics and phosphorylation in primary mouse neurons expressing wild‐type or mutant human tau. (A) Schematic overview of the neuronal model and single‐molecule pull‐down (SiMPull) assay. Primary cortical neurons were transduced with recombinant AAVs encoding either wild‐type (wt) or aggregation‐prone mutant (P301L/S320F) 0N4R human tau and harvested from 8 DIV to 14 DIV. (B) Quantification of soluble tau aggregates using a Simoa assay with an HT7–HT7 sandwich configuration. (C) Levels of total tau (ELISA), HT7‐positive aggregates, and MC1‐positive fibrillar aggregates (Simoa) in mutant tau‐expressing neurons. (D) Representative single‐molecule super‐resolution images of AT8‐positive tau aggregates from wt and mutant neurons across time points. Scale bar: 1 µm. (E) Quantification of aggregate mean length across tau constructs and time points. (F) Proportion of long, fibrillar aggregates (defined as >200 nm length and eccentricity >0.9). (G) Levels of phospho‐tau aggregates (AT8, T181, T217, S356) in mutant neurons, measured by Simoa. Phospho‐tau levels were normalised to HT7 aggregate levels at each time point to estimate the proportion of aggregates positive for each phosphorylation site. Values were further normalized to 8 DIV. Values are plotted as mean ± SD. N = 3 biological replicates with two technical replicates. Panel 2A was created using BioRender.

While post‐mortem human brain tissue provides a static snapshot of the end stage of disease, the aggregation process is extremely dynamic. To overcome this limitation and provide orthogonal validation of candidate interactors identified through the exploratory proteomic screen, we employed a primary cortical neuron culture expressing either wild‐type (wt) or aggregation‐prone mutant (P301L/S320F mt) human 0N4R tau via recombinant adeno‐associated viruses (rAAVs), a well‐established and previously well‐characterized system to model tangle pathology [18]. Neurons were transduced on day 6 in vitro (DIV) and harvested between 8 DIV and 14 DIV (after which neuronal viability declined) for biochemical and imaging analyses (Figure 2A) compared to non‐transduced neurons as a negative control. For each time point, cell culture media was collected alongside cell lysates.

Total protein levels were first quantified using a BCA assay and used to normalize all Simoa and ELISA measurements. Additionally, we measured cytotoxicity by assessing lactate dehydrogenase (LDH) release into the media and examined neuroinflammatory responses using a Simoa‐based ASC speck assay (Figure S4A). We confirmed that no impact of the viral construct on the neuronal health was observed.

To confirm the suitability of this model, we then quantified the proportion of human tau present as aggregates using the tau aggregate Simoa assay (HT7‐HT7) (Figure S4B). These were normalized to the levels of total tau measured using a total tau ELISA. Background levels were observed in control (non‐transduced) neurons at all time points. Wt tau showed a modest increase, reaching 7.6 ± 3.2% aggregation by 14 DIV (from 0.43 ± 0.21% on 8 DIV), while mutant tau displayed a marked and progressive increase in aggregates, peaking at 36.5 ± 14.3% by 14 DIV (from 2.7 ± 1.5% on 8 DIV, Figure 2B). A two‐way ANOVA supported a significant interaction between viral construct and time (*p* < 0.0001), and Tukey post‐hoc tests identified a significant increase in wt and mutant tau aggregates between 8 DIV and 14 DIV (wt: p = 0.0485, mt: p < 0.0001). As expected, no change was observed in the non‐transduced control line.

We also confirmed the time‐dependent progression of tau aggregation in the cultured neurons expressing P301L/S320F mutant human tau by quantifying three distinct tau species: total tau, soluble tau aggregates (HT7‐HT7), and fibrillar aggregates (MC1‐MC1). Total tau levels increased from 481 ± 123 ng mL^−1^ on 8 DIV to 2843 ± 738 ng mL^−1^ on 14 DIV (Figure 2C, wt human tau and negative control in Figure S4B). While HT7+ aggregates showed a significant increase on 12 DIV (8 DIV: 12 ± 6 ng mL^−1^, 12 DIV: 144 ± 83 ng mL^−1^, two‐way ANOVA with Tukey post‐hoc tests, p = 0.0444), MC1 positive tau remained near background until 12 DIV, with a sharp increase by 14 DIV (8 DIV: 12 ± 5 ng mL^−1^, 14 DIV: 475 ± 254 ng mL^−1^, 8 DIV – 14 DIV: p = 0.0478), indicative of a progressive conversion from soluble to fibrillar aggregate forms. This was supported by changes in morphology measured by single‐molecule super‐resolution imaging via single‐molecule pull‐down (SiMPull) with DNA‐PAINT, which enables tau aggregates in the tens to hundreds of nanometers range to be morphologically characterized below the diffraction limit of conventional microscopy. Representative images of aggregates captured from wt neuron lysate using the AT8 antibody (Figure 2D) revealed predominantly small, round aggregates which remained consistently small (68 ± 5.7 nm, 14 DIV; Figure 2E) across the time course. No change was observed in the mean length in wt (two‐way ANOVA with Tukey post‐hoc tests, 8 DIV to 14 DIV: p = 0.0843). In contrast, elongated aggregates consistent with fibrils were found in mutant lysate from 12 DIV onwards, reaching an average length of 193 ± 34.4 nm by 14 DIV (Figure 2E). To more specifically quantify fibrillar aggregates, we defined them based on two morphological features: a length greater than 200 nm and a high eccentricity (>0.9), indicating a highly elongated shape. Using this definition, we found a significant increase in the proportion of fibrillar aggregates in mutant neurons, from 2.7 ± 1.2% on 8 DIV to 17.8 ± 4.0% on 14 DIV (Figure 2F; two‐way ANOVA p < 0.0001 for both tau construct and time, with Tukey post‐hoc t‐test: 8 DIV to 14 DIV wt: p = 0.95, mt: p = 0.0001).

Finally, we examined the phosphorylation profile of the aggregates in the primary neurons expressing P301L/S320F mutant human tau (Figure 2G and Figure S4B) using Simoa assays specific for AT8, pT181, pT217, and pS356 positive aggregates. Each phospho‐epitope exhibited distinct dynamics (Figure 2G); T181‐positive aggregates increased progressively from 8 DIV onwards, AT8 and pS356 showed minimal signal until 10 DIV, and phosphorylation at T217 remained near background until 12 DIV (two‐way ANOVA with Tukey post‐hoc t‐test, 8 DIV to 14 DIV; AT8: p < 0.0001, pT181: p < 0.0001, pT217: p = 0.18, pS356: p = 0.039). To highlight relative temporal changes, each phospho‐tau aggregate signal was normalized to the total HT7‐positive tau aggregates at each time point, estimating the proportion of aggregates that are phosphorylated at each specific site expressed relative to 8 DIV (Figure 2G). This revealed distinct temporal patterns: pT181 exhibited an early peak on 10 DIV before declining, likely due to a larger relative increase in total tau aggregates; pS356 and pT217 showed more stable ratios; and AT8 displayed a continuous increase. These observations support a sequential phosphorylation model, with pT181 appearing earlier than AT8.

To further address whether pT181 and pT217 are preferentially associated with distinct aggregation states, we measured these epitopes in TOMA1‐ and MC1‐captured tau species and normalized each signal to the corresponding TOMA1‐ or MC1‐positive aggregate pool (Figure S5A,B). pT217 showed a relative decline over time, most prominently in MC1‐positive species, whereas pT181 was maintained or increased in TOMA1‐positive species but decreased in MC1‐positive aggregates at 14 DIV. These data indicate that pT181 and pT217 display aggregation‐state‐specific profiles, with reduced relative abundance in MC1‐positive late‐stage aggregates.

### 2.3 Single‐Molecule Co‐Localization Reveals Aggregation‐State‐Specific Tau Interactions

Using this neuronal model, we next sought to determine whether tau‐associated proteins identified in post‐mortem brain tissue showed time‐dependent changes as tau aggregation progressed. The exploratory proteomics analysis identified several candidate proteins with differential association across tau physicochemical states and between AD and control brain tissue, which we prioritized for orthogonal single‐molecule validation and temporal characterization. Based on their recurrent identification as hits in at least three antibody‐defined tau interactomes, we selected ENO1 [19] (a glycolytic enzyme previously shown to correlate with tau PET burden [20]), UBC [21] (ubiquitin; a marker of proteasomal targeting), SLC1A6/EAAT4 (a neuronal glutamate transporter), APP (the precursor of amyloid‐β), and APOA1 (an apolipoprotein involved in lipid transport). hnRNPA1 [22], an RNA‐binding protein implicated in stress granule formation and neurodegeneration, was selected because its differential abundance in all tau‐depleted supernatants, suggesting a more transient interaction. HSPA1B/Hsp70‐2 (a molecular chaperone involved in proteostasis, hereafter referred to as Hsp70), was additionally included as a biologically informed [23] candidate despite being identified as a hit in only one tau interactome. We also examined Aβ42‐specific association to distinguish it from the broader APP/Aβ signal. MAP2, whose association with TOMA1‐captured tau was reduced in AD, was included for comparison.

**FIGURE 3:**
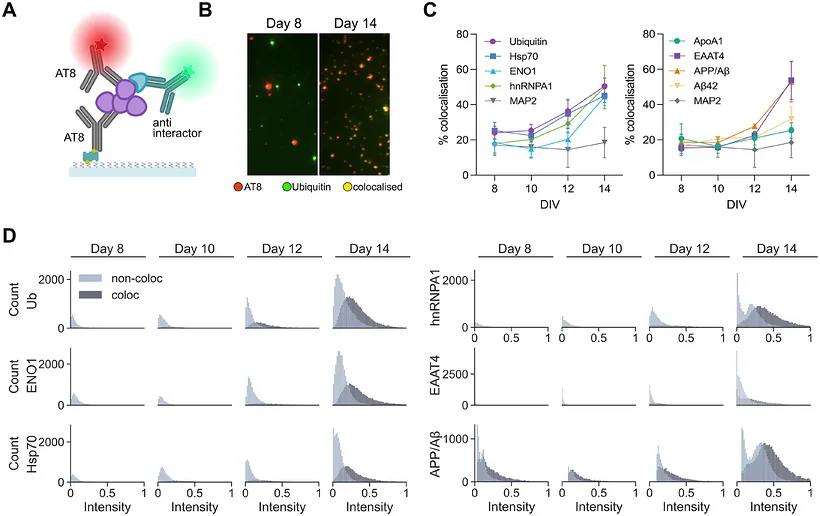
Single‐molecule co‐localization reveals dynamic, size‐dependent tau–protein interactions during aggregation. (A) Schematic of SiMPull co‐localization assay. Tau aggregates were captured using the AT8 antibody and detected using AF647‐labelled AT8 and AF488‐labelled antibodies against candidate interactors. (B) Representative dual‐color single‐molecule images showing co‐localization of AT8 (red) and ubiquitin (green) on 8 DIV and 14 DIV. Yellow puncta indicate co‐localized aggregates. Scale bar: 1 µm. (C) Quantification of the percentage of AT8‐positive aggregates co‐localizing with each interactor across time points. Values are plotted as mean ± SD. N  =  3 biological replicates with two technical replicates. Interactors split for visual purposes. Aβ42: 12F4 antibody. APP+Aβ: 6E10 antibody. (D) Histogram distributions of normalized fluorescence intensity (a proxy for aggregate size) for co‐localized (dark grey) and non‐co‐localized (light grey) AT8 aggregates at each time point.

We examined the association of each interactor with individual aggregates using a SiMPull approach coupled with dual‐color fluorescence imaging (Figure 3A). Tau aggregates were captured using the AT8 antibody, and then simultaneously detected with fluorescently labelled AT8 (AF647) and respective candidate interactor antibodies (AF488). We used AT8 here as it captured the time‐dependent progression (Figure 2D–G), and it was positive for most of the candidate interactors. This strategy allowed us to specifically quantify the co‐localization of candidate interactors with individual tau aggregates without the need for conformation‐specific antibodies so that we would capture aggregates at all aggregation states. Representative images (Figure 3B) demonstrate an increase in co‐localization between AT8 and ubiquitin from 8 DIV to 14 DIV, a trend that was observed for all candidate interactors (Figure 3C). Quantitative analysis revealed on average only 19.4 ± 5.7% of tau aggregates were decorated with each interactor on 8 DIV, which progressively increased from 10 DIV onwards (Figure 3C), reaching a maximum of 45.1 ± 3.8% for Hsp70 by 14 DIV. The number of aggregates containing ubiquitin and Hsp70 rose linearly from 10 DIV, while hnRNPA1 lagged behind until 12 DIV. Similarly, ENO1 did not significantly increase until 14 DIV, suggesting it preferentially associates with more late‐state tau aggregates (Figure S6). EAAT4 and APP/Aβ species (detected through 6E10 antibody) similarly showed marked late‐stage increases in co‐localization, coinciding with the emergence of larger and more fibrillar tau aggregates. By contrast, Aβ42‐specific co‐localization increased more modestly, indicating that the broader APP/Aβ signal detected using 6E10 is unlikely to be explained by Aβ42 alone. APOA1, whose association with tau was reduced in AD compared with control brain tissue in the proteomics analysis, showed little change across the neuronal time course as expected.

Lastly, we investigated an additional interactor whose association with tau was reduced in AD brain tissue. MAP2, a microtubule‐associated protein, showed decreased association with oligomeric tau (TOMA1) in the proteomics experiment. In the single‐molecule assay, the MAP2 signal was readily detectable but showed minimal co‐localization with AT8‐labelled aggregates at any time point. A small, increasing degree of co‐localization was observed with HT7‐captured tau species, suggesting that MAP2 preferentially interacts with tau before it becomes phosphorylated and aggregated (Figure S7A,B).

To test whether the association of these interactors with tau aggregates is dependent on aggregate size, we used intensity as a proxy for aggregate size [13, 24]. To support this interpretation, defined synthetic multiepitope tau constructs were tested, showing progressively increasing fluorescence intensity from monomer to tetramer, while mean SiMPull brightness was positively correlated with mean aggregate length measured independently by DNA‐PAINT across matched samples (Figure S8A,B). We then examined the fluorescence intensity distribution of AT8 aggregates stratified by interaction status (co‐localized vs non‐co‐localized) (Figure 3D) and found that co‐labeled tau aggregates consistently showed higher intensities than non‐labeled ones, indicating these interactors are preferentially associated with larger or more mature aggregates. This trend was amplified over time. Notably, Hsp70 co‐localization emerged earlier (10 DIV), consistent with its role as a chaperone recognizing early misfolded tau species. In contrast, ENO1 associated almost exclusively with brighter, late‐state aggregates, supporting its potential role in the broader metabolic and proteostatic dysfunction seen in advanced tauopathy [20].

To further examine whether selected tau interactors associate with structurally distinct aggregate subsets, and whether their binding represents transient surface decoration or a more stable association, we performed Proteinase K digestion for 0 – 30 min on pooled AD brain homogenate followed by co‐localization SiMPull for ENO1, Hsp70, ubiquitin and hnRNPA1 (Figure S9A). Proteinase K treatment reduced levels of both tau aggregates and their interactors, consistent with progressive proteolysis. However, for ENO1, Hsp70 and ubiquitin, the remaining co‐localized (interacting) aggregates were markedly brighter than non‐colocalized aggregates (Figure S9B). This enrichment increased with digestion time, indicating that these proteins remain associated with a subset of larger, more protease‐resistant aggregates. By contrast, hnRNPA1 signal fell to background within 10 min of digestion, with very few co‐localized aggregates remaining, consistent with a weaker, surface‐exposed interaction. Together, these results indicate that tau–protein interactions differ in their structural context and proteolytic stability, ranging from stronger association with protease‐resistant assemblies to more transient interactions.

### 2.4 Inferring a Pseudotemporal Trajectory of Tau Aggregation in Human Brain

**FIGURE 4:**
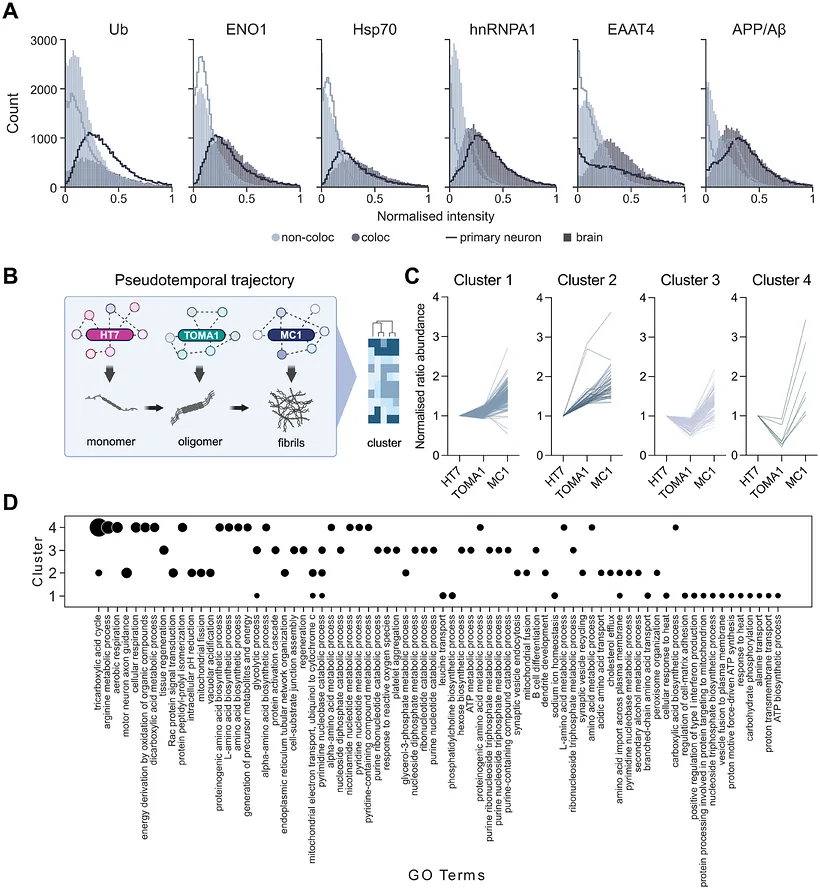
Pseudotemporal clustering and functional characterization of tau interactors across aggregation states in AD brain. (A) Single‐molecule co‐localization intensities of AT8‐tau aggregates interacting with Ubiquitin, ENO1, Hsp70, hnRNPA1, EAAT4 or APP/Aβ in post‐mortem human brain (*n* = 5 AD, *n* = 5 HC). The brightness histograms from the mouse primary neurons are shown as lines for reference. (B) Schematic of pseudotemporal clustering of the interactors based on aggregation progression (HT7, mostly monomeric – TOMA1, oligomeric – MC1, aggregate). (C) Mean HT7‐normalised abundance trajectories of each protein by cluster across tau aggregation states (from HT7 to TOMA1 to MC1). (D) Gene ontology enrichment in each cluster identified in C. Dot size corresponds to fold enrichment, and clusters are arranged by pseudotemporal progression. Panel 4B was created using BioRender.

Single‐molecule imaging confirmed that the size of AT8‐positive tau aggregates in post‐mortem brain extracts closely mirrored those observed at the final time point in neurons expressing mutant human tau (Figure 4A, histogram: human brain, lines: primary neurons). Across ubiquitin, ENO1, Hsp70, hnRNPA1, EAAT4 and APP/Aβ, co‐labelled aggregates were consistently brighter (≈larger) than non‐co‐labeled aggregates in both systems, reinforcing the accuracy of the neuronal model in recapitulating relevant aggregate phenotypes present at the end stage of disease.

To characterize how selected interactor associations varied across tau species in human brain, we quantified the proportion of tau aggregates (labelled with AT8, HT7, or MC1) that co‐localized with selected interactors in AD samples, normalized to age‐matched control samples (Figure S10). AT8‐positive aggregates showed the highest relative association with ENO1 and ubiquitin, followed by hnRNPA1 and Hsp70. HT7‐positive aggregates showed higher interaction with hnRNPA1 and Hsp70 but no significant difference for ENO1 and ubiquitin. MC1‐positive aggregates showed increased association with all three proteins, consistent with the trends observed in the primary neurons.

These observations further support the physiological relevance of the neuron model and indicate that tau–protein interactions change as aggregates mature in human disease. To capture this continuum directly in human tissue, we leveraged the aggregation‐state‐specific antibodies in our proteomics panel to reconstruct a pseudotemporal trajectory of the tau aggregate interactome. We note that these antibodies are not strictly state‐exclusive, and therefore the resulting interactomes should be interpreted as antibody‐enriched tau‐associated protein profiles rather than discrete conformational classes. To support this framework, we performed orthogonal validation against monomeric tau, small tau aggregates, heparin‐assembled aggregates and fibrils isolated from Alzheimer's disease brain tissue confirmed the expected differential reactivity of HT7, TOMA1 and MC1 across these species (Figure S1). We therefore used HT7 to represent predominantly monomeric species, TOMA1 to capture small aggregates (oligomeric species), and MC1 to reflect late‐stage fibrillar assemblies.

Using this framework, we examined how interactor abundance shifted across successive aggregation states in human brain tissue (Figure 4B–D). The abundance of each interacting protein was normalized to its level in the HT7 pulldown, allowing us to consider fold changes between successive states (HT7 to TOMA1 and TOMA1 to MC1; Figure S11A). Hierarchical clustering of these relative abundances revealed four distinct clusters (Figure 4C and Figure S11A). Clusters 1 and 2 consistently increased in their association with maturing tau aggregates, while clusters 3 and 4 both decreased in association with oligomeric tau before showing sharp increases in their association with fibrillar tau (Figure 4C).

To infer the potential functional relevance of these profiles, GO enrichment analysis was applied to the proteins associated with each cluster (Figure 4D). Proteins in cluster 1, stably associated across all tau conformers, were associated with core metabolic pathways such as mitochondrial electron transport and phosphatidylcholine biosynthesis, suggesting constitutive cellular interactions with tau. Cluster 2, which increased toward the oligomeric state, was enriched for organelle organization and early stress‐response pathways (e.g., GO:0071786 endoplasmic reticulum tubular network organization, GO:0007031 peroxisome organization) consistent with an early stress response to misfolded tau species, and synaptic impairment (GO:0036465 synaptic vesicle recycling, GO:0099643 signal release from synapse). Proteins in cluster 3 were enriched for RNA metabolism (GO:0009261 ribonucleotide catabolic process), nucleotide catabolism (GO:0006208 pyrimidine nucleobase catabolic process), and glycolysis (GO:0006096 glycolytic process). Cluster 4, specific to the MC1 pulldown, contained only 7 proteins, particularly mitochondrial energy metabolism and redox balance (ACO2, CS, MDH1, GLUL, GO:0046496 nicotinamide nucleotide metabolic process), as well as a stress response (PPIA, DDAH1, GO:0000413 Protein peptidyl‐prolyl isomerization) and MAPT (likely reflecting increased self‐association of tau at the fibrillar state). Their specific enrichment in the MC1 pulldown links late‐state fibrillar tau aggregates with proteins involved in neuronal metabolism and homeostasis. These associations could reflect either sequestration of essential enzymes, toxic gain‐of‐function or a downstream response to pathology.

To test whether these patterns generalize to other physicochemical features of tau aggregates known to progress with time, we performed a parallel analysis using the phosphorylation‐specific antibody panel (HT7 to T181 to AT8), reflecting the temporal sequence observed in our neuronal model while recognizing that this ordering may not apply uniformly to human disease (Figure S11B,C). Despite targeting a different marker of pathology, clusters displayed similar functional shifts (early‐state enrichment for mitochondrial processes and cytoskeletal organization, and late‐state enrichment for RNA metabolism, nucleotide catabolism and glycolysis), consistent with convergence of the aggregation and phosphorylation pathways in human tissues.

Together, these findings provide evidence that tau‐protein interactions evolve with aggregate maturation, with distinct proteins associating preferentially with early or late‐state aggregates. The proteome‐wide trajectories should be interpreted as hypothesis‐generating, whereas selected candidate interactions are validated by orthogonal single‐molecule measurements. Importantly, this pseudotemporal approach connects our knowledge of in vitro aggregation dynamics, accessible using model systems, with end‐stage tau profiles observed in human brain tissue to provide a more holistic view of tau pathology.

## 3 Discussion

This study characterized how the tau interactome changes throughout the course of aggregation, from monomers to large fibrils. By integrating exploratory proteomic analyses and single‐molecule imaging in both a primary neuron model and post‐mortem human brain tissue, a dynamic remodeling of the tau interactome, as aggregates mature, was revealed. We identified temporally distinct functional interactome modules and correlate this with a pseudotemporal framework that enables the evolution of the tau interactome with aggregation to be explored in human tissue extracts. This approach yields new insights into molecular processes associated with tau‐mediated pathology.

To span distinct tau conformational states in the proteomics experiment, we used a panel of antibodies targeting total, oligomeric, fibrillar, and phosphorylated species. While conformation‐specific antibodies such as TOMA1 can be limited by uncertain epitope specificity [25], the SiMPull approach employs a sandwich detection format to ensure robust and specific aggregate capture regardless of conformation. The single‐molecule pulldown strategy then enabled direct visualization of tau aggregates and their associated proteins with single‐aggregate resolution. By capturing aggregates onto a coverslip and applying dual‐color imaging, it was possible to discriminate aggregate size and interaction status. These parameters are difficult to resolve using bulk proteomics or proximity‐based methods such as ligation assays [26], biotinylation (e.g., BioID, APEX) [27], or FRET [28]. This integrative platform provides a powerful and complementary tool for dissecting tau–protein interactions at the level of individual aggregates and across aggregation stages.

Using a cell culture model of neurons expressing P301L/S320F human tau, we observed a clear temporal progression of tau aggregation. Total tau accumulated steadily over time while the formation of aggregates detected using HT7 and MC1 was delayed, becoming pronounced only at later time points. This lag highlights a window where tau remains largely monomeric, likely recapitulating the earliest phases of disease where the dysfunctional tau must reach a critical concentration before aggregates are detectable. Morphological analysis confirmed a transition from small, round aggregates to elongated fibrils as expected. In addition, Simoa analyses confirmed phosphorylation of tau aggregates at T181 preceded AT8, supporting a sequential phosphorylation model consistent with prior studies [29, 30]. While these previous studies have inferred ordered phosphorylation events from bulk tau measurements, the aggregate‐specific approach enables a more precise, quantitative characterization of post‐translational modification dynamics during aggregate formation. Nevertheless, overexpression of aggregation‐prone P301L/S320F tau is likely to accelerate aggregation kinetics and may also enhance interactor recruitment relative to physiological human disease. These data provide aggregate‐specific temporal information into how tau conformational and post‐translational changes unfold in parallel within the neuronal model.

GO analysis revealed functional remodeling of the tau interactome across aggregation states. At the predominantly monomeric state represented by HT7, interactors were associated with proteolytic quality control mechanisms, including ER stress and autophagy pathways, alongside immune activation signatures, consistent with engagement of proteostatic and immune‐related pathways during early tau dysregulation. Oligomeric tau aggregates (captured using TOMA1) showed increased association with regulators of cytoskeletal organization, neuronal morphogenesis, and synaptic processes, with concurrent reduction in the association of proteins linked to metabolic pathways. These findings are consistent with previous reports highlighting early activation of the unfolded protein response in AD [31] and suggest that disruptions to cytoskeletal integrity emerge early in response to oligomeric tau accumulation [32]. This intermediate stage may reflect an inflexion point where neurons initiate compensatory remodeling in response to early pathology. Notably, most differentially recovered TOMA1‐associated proteins were reduced in AD. This may reflect biological remodeling or loss of interactions at the oligomeric tau state; however, technical factors, including altered TOMA1 epitope accessibility, epitope masking within larger or compositionally distinct aggregates, or differences in immunoprecipitation efficiency, may also contribute. Mature tau aggregates (MC1) were enriched for cytoskeletal and postsynaptic components, with mixed up‐ and down‐regulation of proteolysis, consistent with widespread structural disruption and potential failure of degradation mechanisms.

Previous mechanistic work from our group showed that Hsp70 can inhibit tau nucleation and elongation and sequester potentially toxic and seeding‐competent aggregates, suggesting that its early recruitment may reflect an attempted protective response [33]. Similarly, N‐terminal monoubiquitylation of tau can alter its assembly and facilitate proteasome‐mediated oligomer removal [34]. However, the consequences of ubiquitin recruitment depend on the modified substrate, conjugation site, ubiquitin‐chain architecture and available clearance machinery. Thus, the associations observed here do not establish whether these proteins promote clearance, alter aggregate assembly or accumulate following unsuccessful proteostasis.

This work complements recent proximity‐labeling studies such as the ProPPr analysis, which mapped phospho‐tau interactomes across tauopathies [35]. While that study provided a valuable cross‐sectional snapshot of disease subtypes, our approach combines temporal information from the neuronal model with aggregation‐state‐resolved profiling in human tissue, providing insight into how tau–protein associations may change as aggregates mature.

The integration of single‐molecule imaging in this study is an important advance; it allows the quantitative evaluation of the association of individual interactors with specific tau aggregate types. These analyses demonstrated that tau–protein interactions vary with aggregate maturation, with co‐localization of many interactors seen more frequently for larger, brighter aggregates consistent with size‐dependent association. Similar trends were observed in both neurons and AD brain tissue, and this information cannot be obtained from proteomics alone.

Beyond this, our data indicate that interacting aggregates represent a distinct subset: they were consistently brighter (indicating larger or epitope‐dense structures) and, for several interactors, more resistant to Proteinase K digestion, suggesting preferential binding to larger/more compact and protease‐stable tau assemblies. Together, these observations suggest that small tau aggregates can exist within stable tau‐associated protein complexes comprising a diverse set of interacting proteins. While these data do not establish whether interactors are structurally incorporated into tau assemblies or whether they remain associated through stable but non‐stoichiometric interactions, the stability of these interactions upon dilution and their resistance to protease digestion suggest that these are relatively stable interactions, rather than just transient. This raises the possibility that interactions with chaperones, degradation machinery, and other cellular proteins may influence the biochemical properties or maturation trajectory of tau aggregates. Association with non‐tau components could, for example, sterically hinder ordered fibril elongation and may help explain the absence of fibrillar morphology in many small aggregates in the brain, although this mechanism was not directly tested here. On this basis, we propose a model in which early tau aggregation is accompanied by the formation of mixed tau–protein assemblies that may represent an initial cellular attempt to buffer misfolded tau. Aggregate maturation may then reflect a progressive failure or saturation of these protective mechanisms, leading to the accumulation of larger, more protease‐resistant structures enriched for cytoskeletal and synaptic components. This model frames tau aggregation as a multi‐component process shaped by cellular responses to proteostatic stress, rather than a simple, tau‐autonomous fibrillization pathway.

Together, these data support distinct aggregation state‐dependent association patterns for key tau interactors. Hsp70, a molecular chaperone, associated early and persisted in larger aggregates, consistent with engagement of chaperone‐mediated proteostasis that may become overwhelmed or saturated over time. This aligns with previous work showing that Hsp70 can bind tau aggregates with high affinity [23]. ENO1, a glycolytic enzyme, was associated predominantly with more mature tau aggregates potentially reflecting metabolic dysfunction in late‐stage disease, consistent with PET imaging studies linking ENO1 to increased tau burden in AD [20]. Ubiquitin, which was present across all states but enriched in later aggregates, may indicate persistent but ineffective proteasomal targeting of pathological tau. The increasing association of hnRNPA1 with tau aggregates may reflect early‐stage interactions, as hnRNPA1 has been observed to co‐localise with neurons containing mid‐stage tau inclusions but not with larger neurofibrillary tangles, suggesting a role in the mid‐phases of tau aggregation [22]. This is consistent with previous work showing that tau inclusions turn over rather than remaining static structures, indicating active clearance pathways [18]. The increasing association of ubiquitin and Hsp70 in later aggregation states may therefore reflect clearance mechanisms that become progressively less effective as aggregates mature and accumulate. These interpretations are consistent with the known functions of these proteins but remain hypotheses; the present data establish temporal associations and do not determine whether individual interactors causally influence tau aggregation.

The modest size of the post‐mortem human brain cohort has limited statistical power and may not fully capture the substantial biological heterogeneity of AD. The proteomic analysis was therefore treated primarily as a hypothesis‐generating discovery screen, used to nominate candidate aggregation‐state‐dependent tau interactors and pathway‐level patterns rather than to provide confirmatory evidence based on statistical significance alone. Using this approach led us to a set of interactors that were successfully confirmed by orthogonal single‐molecule imaging methods. The consistency between the neuronal model and human brain tissue further supports the robustness of the main biological conclusions. While the primary mouse neuron model expressing human tau cannot fully recapitulate the human disease environment, it offers key advantages in terms of temporal control and reproducibility. The consistency between the neuronal model and human tissue at the end‐stage supports its physiological relevance and provides a basis for relating the temporal changes observed in the model to aggregation‐state‐associated patterns in the human brain. Nevertheless, the model lacks the complex cellular environment of the human brain, including glial responses and co‐pathologies such as Aβ, which may also modulate tau–protein interactions in vivo [36]. Integrating these findings with multicellular systems, such as organoids or co‐culture models, may provide further insight into how these factors shape the tau interactome across disease contexts. In this study, AT8 was selected because it is a widely established marker of pathological tau, including in neuropathological staging, it recognizes a composite epitope in the S202/T205 region and therefore does not distinguish phosphorylation at these individual sites. Future studies using site‐specific antibodies could determine whether pS202‐ or pT205‐positive tau species show distinct aggregate‐state‐specific profiles, and how these compare with tau species carrying the combined AT8 phospho‐epitope. Although the robustness analyses support the stability of the pseudotemporal clustering, the framework simplifies a likely continuous and heterogeneous aggregation process into a small number of overlapping antibody‐enriched profiles. It should therefore not be interpreted as evidence that tau progresses through discrete or obligatory sequential biological states. Lastly, a limitation of using pull‐down‐based approaches is that these may preferentially capture tau species with accessible epitopes and may not fully preserve weak or conformation‐sensitive interactions. However, as all samples were processed under matched conditions, these effects are expected to be consistent across groups, supporting the comparative interpretation of antibody‐defined tau interactomes. Label‐free approaches such as atomic force microscopy could provide complementary topographical information on aggregate morphology, including height, length and fibrillar features, although such approaches would not provide the molecular specificity required to define tau aggregate‐associated protein interactions in complex biological samples.

Together, these findings illustrate how tau aggregation is accompanied by progressive changes in the association of proteins involved in proteostasis, metabolism, cytoskeletal organization, and synaptic function. By resolving these interactions across aggregation states, our data highlight molecular pathways that may be differentially engaged during early and late phases of tau pathology and provide candidates for future functional investigation. These stage‐associated interaction patterns raise the possibility that different phases of tau pathology may require distinct intervention strategies, although functional studies will be needed to determine whether individual pathways can modify aggregate formation or toxicity. More broadly, this integrative strategy – combining proteomics with single‐aggregate imaging – offers a framework for mapping dynamic protein interactions in other proteinopathies and informing future stage‐specific diagnostic and therapeutic studies.

## 4 Conclusion

Proteomics is a powerful technique for mapping protein‐protein interactions and understanding how they change in disease. However, as it is inherently a bulk approach, it provides population‐averaged data that may obscure molecular heterogeneity at the single‐protein level. To address this limitation, proteomics can be complemented by single‐molecule techniques, which allow for the characterization of individual protein complexes with high spatial and temporal resolution. Within this framework, the proteomic analysis serves as a hypothesis‐generating discovery screen, while single‐molecule measurements provide orthogonal validation of selected candidate associations. The integration of these approaches provides a more comprehensive view of protein interactions, capturing both large‐scale interactome changes and the specific molecular interactions linked to pathological processes. This combined strategy can help identify stage‐specific or aggregation‐state‐specific interaction signatures, informing the development of targeted interventions or biomarkers based on tau's evolving molecular interactions.

## 5 Methods

### 5.1 Human Post‐Mortem Brain Tissue Details

Post‐mortem brain tissue from five donors with AD and five age‐matched control donors was obtained from the Edinburgh Brain Bank with the approval from the East of Scotland Research Ethics Service (REC 21/ES/0087). Written informed consent from the donors and/or their relatives was provided as appropriate. Donor characteristics are summarized in Table 1, for which no significant differences were identified between each cohort (AD vs HC).

### 5.2 Homogenisation of Post‐Mortem Brain Tissue

The post‐mortem brain tissue samples were obtained from the Edinburgh Brain Bank, where they were flash‐frozen and stored at −80°C. The brain tissue samples were homogenized as previously described [13]. Briefly, tissue samples were homogenized using a VelociRuptor V2 Microtube Homogenizer (Scientific Laboratory Supplies, Cat. No. SLS1401) at 5 m/s for two 20‐second cycles, with a 10‐second pause between cycles, at 4 °C. Homogenization was performed in 10 volumes of ice‐cold buffer (10 mM Tris‐HCl, 0.8 M NaCl, 1 mM EGTA, 0.1% Sarkosyl, 10% sucrose; pH 7.32), supplemented with complete Ultra Protease Inhibitor and PhosStop Phosphatase Inhibitor. The homogenate was centrifuged at 21 000 × g for 20 min at 4°C, and the upper 90% of the supernatant was collected. The remaining pellet was re‐homogenized in 5 volumes of the same buffer and centrifuged again under the same conditions. The upper 90% of this second supernatant was pooled with the first. Combined supernatants were aliquoted and stored at −80°C for subsequent analysis. Total protein concentration was measured using the BCA assay (Thermo Fisher, Cat. No. 23227), following the manufacturer's protocol. Total tau levels were determined using the Tau ELISA kit (abcam, Cat. No. ab273617) according to the manufacturer's instructions.

### 5.3 Proteinase K Digestion

AD brain homogenate samples from 5 patients were pooled and incubated with 1 µg mL^−1^ Proteinsase K (New England Biolabs) at 37°C for 0, 10, 20, or 30 min before PMSF was added to a final concentration of 1 mM. The samples were then incubated for 10 min on ice. The final brain homogenate dilution was 1:10 for SiMPull analysis.

### 5.4 Preparation of rAAVs

rAAV2/TM8 [38] to express wt human tau (WT‐htau0N4R) or pro‐aggregant mutant human tau (P301L/S320F‐htau0N4R) under the control of the Synapsin promoter was prepared as microscale rAAVs. HEK293T cells (ATCC) were maintained as recommended in DMEM (Gibco, Life technologies) with 10% (vol/vol) FBS (Gibco, Life Technologies), 2 mM l‐glutamine, and 1% (vol/vol) penicillin/streptomycin (Thermo Fisher Scientific). HEK293T cells were plated in 15 cm dishes in 25 mL media 24 h before transfection of transgene plasmid with TM8 capsid (AAV2/8‐3Y) and pHelper using Polyethylenimine Linear (Polysciences) at 60–80% confluency. 24 h following transfection, the media was changed to serum‐free Optimem (Gibco, Life Technologies) with 1% v/v penicillin/streptomycin. The microscale virus was harvested 48 h after switching to Optimem by centrifuging the media at 500 g for 5 min and collecting the supernatant. The supernatant was concentrated by centrifugation at 4000 rpm using Amicon 15 100 000 MWCO concentration units (Millipore, UFC910024). Genomic titers (viral genomes (VGs) per mL) were confirmed via quantitative real time PCR (qPCR) with probes against the ITRs as previously described by Goodwin et al. [38]. Concentrated media containing secreted rAAVs (microscale virus) were then applied to primary neurons by adding rAAVs directly into the culture medium at 2.5 × 10^10^ VG mL^−1^.

### 5.5 Primary Mouse Cortical Neurons

Primary mouse cortical neurons were prepared from the cortex of wild type CD1 embryos at E16.5 as described in Yang et al. [39]. and Böken et al. [24]. Briefly, brains were harvested in ice‐cold dissection buffer containing 1 mM HEPES in HBSS, the meninges removed, and the cerebral cortices isolated. Following mechanical dissociation, cells were seeded at a density of 250.000 cells well^−1^ in 12‐well plates pre‐coated with 10 µg mL^−1^ polyD‐lysine (Sigma–Aldrich, P7280) and cultured in Neurobasal medium (Gibco, 21103049) supplemented with 2% B27 (Gibco, 17504044), GlutaMAX (Gibco, 35050061) and penicillin (100 U mL^−1^) and streptomycin (100 µg mL^−1^; Gibco, 15140122). Cells were placed in a humidified incubator at 37°C in 5% CO_2_. At 6 DIV, neurons were transduced with adeno‐associated viruses (AAVs) to express wt (AAV2/TM8‐WT‐htau0N4R) or mutant (AAV2/TM8‐P301L/S320F‐htau0N4R) human tau at 2.5 × 10^10^ viral genome (VG)/mL. Non‐transduced cells served as a negative control. At 8, 10, 12 and 14 DIV cell culture media was collected, and neurons were harvested in lysis buffer containing 50 mM Tris‐HCl, 150 mM NaCl, 1% Triton X‐100, and 5 mM EDTA followed by centrifugation at 10 000 × g for 5 min at 4°C. Each independent experiment is defined by an independent neuronal preparation and included two technical replicates (two wells) per timepoint and viral construct. All conditions were grown in three biological replicates, each including two technical replicates.

Total protein concentration was measured using the BCA assay (Thermo Fisher, 23227), following the manufacturer's protocol. Total tau levels were determined using the Human Tau ELISA kit (abcam, ab273617) according to the manufacturer's instructions. LDH was measured using the LDH Assay Kit (abcam, ab65393), following the manufacturer's protocol.

### 5.6 Antibodies

All antibodies were obtained as a carrier‐protein free composition. All tau antibodies were monoclonal to enable specific aggregate detection. The antibodies used in the study are listed in Table 2.

**TABLE 2:** Overview of antibodies employed in the study. MC1 antibody is not commercially available and was available through the Feinstein Institutes for Medical Research (“Feinstein”), on behalf of Albert Einstein College of Medicine. RRID = Research Resource Identifiers. SR = super‐resolution microscopy experiment.

	Antibody	Manufacturer	Cat. No.	RRID	Clonality	Reactivity	Simoa	Co‐loc	SR
Tau	HT7	Invitrogen	MN1000(b)	AB_2314654	mono	H	x	x	x
	TOMA1	Sigma‐Aldrich	MABN819	AB_3068608	mono	H, M	x	x	
	MC1	Feinstein	N/A	AB_2314773	mono	H, M	x	x	
	AT8	Invitrogen	MN1020(b)	AB_223647	mono	H, M	x	x	x
	T181	abcam	ab236458	AB_3754142	mono	H	x		
	T217	abcam	ab314558	AB_3754143	mono	H	x		
	S356	abcam	ab196367	AB_3754144	mono	H	x		
Interactors	Hsp70	abcam	ab219597	AB_3754145	mono	H, M		x	
	hnRNPA1	abcam	ab5832	AB_305145	mono	H, M		x	
	Ubiquitin	abcam	ab134953	AB_2801561	mono	H, M		x	
	ENO1	abcam	ab229378	AB_3754146	mono	H, M		x	
	MAP2	Invitrogen	PA1‐16751	AB_2138189	poly	H, M		x	
	EAAT4	Novus Bio	NBP3‐13357	AB_3588222	poly	H, M		x	
	APOA1	abcam	ab52945	AB_2056661	mono	H		x	
	6E10	BioLegend	803007	AB_2715854	mono	H		x	
	12F4	BioLegend	805501	AB_2564683	mono	H, M		x	
Misc	AL177	AdipoGen	AL177	AB_2490441	poly	H, M			x

***Fluorophore labeling***: Carrier protein‐free antibodies were fluorescently labelled with AF647 or AF488 using the Alexa Fluor Conjugation Kit (Fast)—Lightning‐Link (Abcam, ab269823 and ab236553) according to the manufacturer's instructions. Excess fluorophore was removed using a 40 kDa Zeba Spin desalting column (Thermo Fisher, A57756) followed by a 50 kDa Amicon Ultra spin column (Merck, UFC5050). Dye‐labelled antibodies were stored at 4°C. Protein concentration and degree of labelling were determined by absorption at 280 and 494 nm (AF488) or 650 nm (AF647), correcting for dye absorption at 280 nm. The degree of labelling was within the manufacturer's recommended range of 3–9 dyes per antibody.

***Biotin labeling***: Antibodies were biotinylated using the Biotin Conjugation Kit (Fast, Type B)—Lightning‐Link (Abcam, ab201796) according to the manufacturer's instructions. Excess biotin was removed using a 40 kDa Zeba Spin desalting column (Thermo Fisher, A57756) followed by a 50 kDa Amicon Ultra spin column (Merck, UFC5050). Biotin‐conjugated antibodies were stored at 4°C. Protein concentration was determined by absorption at 280 nm.

***DNA labeling***: For DNA‐PAINT experiments, DBCO‐conjugated docking strand (DBCO‐concatDS2: DBCO‐TEG‐AAACCACCACCACCACCACCACCACCACCACCACCA) and the Atto655‐labelled imaging strand (TGGTGGT‐AminoC7‐Atto655) were obtained from ATDBio (Southampton, UK). Lyophilized oligonucleotides were dissolved in 18.2 MΩ cm water (0.02 µm‐filtered; VWR, 516–1501), concentrations were determined by A260, and aliquots were stored at −20°C. Antibodies were functionalized with the docking strand using the SiteClick Antibody Azido Modification Kit (Invitrogen, S20026) following the manufacturer's instructions. Briefly, azido‐modified antibodies were incubated overnight at 37°C with 10 molar equivalents of docking strand. Excess docking strand was removed using a 100 kDa Amicon Ultra spin column (Merck, UFC510024). Antibody concentration and labelling efficiency (typically 3–4 docking strands per antibody) were determined by A280 and A260/A280.

### 5.7 Simoa Reagent and Plate Preparation

***Bead coupling***: Antibody‐bead conjugation was performed following the manufacturer's protocol (Quanterix) and as previously described [40]. Carboxylated magnetic beads were washed, activated with EDC (0.3 mg mL^−1^) for 30 min at 4°C, and incubated with buffer‐exchanged antibody (0.2 mg mL^−1^) for 2 h at 2–8°C. Following incubation, beads were washed, blocked, and stored at 4°C until use. Coupling efficiency was assessed by measuring unbound antibody in the supernatant through absorption at 280 nm.

***Plate preparation***: All assays were run using the 3‐step assay protocol (Quanterix) as previously described [40]. Samples were diluted with Tau 2.0 sample diluent and added to a 96‐well plate along with ∼500 000 antibody‐coupled beads. Plates were incubated for 30 min at 30°C with shaking at 800 rpm, followed by washes and incubation (10 min, 800 rpm, 30°C) with a biotinylated detector antibody (0.3 µg mL^−1^), and then with streptavidin‐β‐galactosidase (SBG; 150 pM, 10 min, 800 rpm, 30°C). Beads were washed, dried, and processed on the SR‐X platform.

***Simoa processing***: On the SR‐X Instrument, beads were resuspended in RGP substrate and loaded into microwell arrays on the Simoa Disc. The fraction of active wells (f_ON_) was calculated automatically and used to calculate the average enzymes per bead (AEB) assuming Poisson statistics. Conditions exceeding f_ON_ > 0.9 were avoided to maintain digital signal detection. A sample of cell‐derived hyperphosphorylated tau aggregates of known concentration [13] was used as a standard. After obtaining the AEB values at each calibration level, a four‐parameter logistic (4PL) curve was fitted.

### 5.8 SiMPull

***Coverslip passivation***: Coverslips were prepared for SiMPull as previously described [13] using a method adapted from Chandradoss et al. [41]. Briefly, #1.5 borosilicate glass coverslips (26 × 76 mm; VWR, Cat. No. MENZBC026076AC40) were sequentially sonicated for 10 min each in 18.2 MΩ·cm water, acetone, and methanol, followed by 20 min in 1 M KOH. After rinsing with water and methanol, coverslips were dried with nitrogen and plasma‐cleaned for 15 min (Femto Plasma Cleaner; Diener Electronic). Coverslips were then silanized in a 3:5:100 solution of APTES (Fisher Scientific, Cat. No. 10677502), acetic acid, and methanol. This involved 1 min sonication, 10 min static incubation, a second 1 min sonication, and a final 10 min incubation. Coverslips were rinsed twice with water and once with methanol, dried with nitrogen, and fitted with 50‐well silicone gaskets (Grace Bio‐Labs, SKU 103250). For passivation, 9 µL of a freshly prepared 100:1 mixture of SVA‐PEG‐OMe (110 mg mL^−1^, Mw ∼5000; Laysan Bio) and SVA‐PEG‐Biotin (100 mg mL^−1^, Mw ∼5000; Laysan Bio) was added to each well, followed by 1 µL of 1 M NaHCO_3_ (pH 8.5). Coverslips were incubated in a humidity chamber for 24 h, rinsed twice with water, and dried with nitrogen. A second passivation step followed using 9 µL of methyl‐PEG4‐NHS ester (10 mg mL^−1^; Thermo Fisher, Cat. No. 22341) and 1 µL of 1 M NaHCO_3_. After another 24 h incubation, coverslips were rinsed with water, dried with nitrogen, and stored desiccated at –20°C until use.

***SiMPull for co‐localization***: SiMPull was performed as previously described [13]. Wells were first washed once with PBST (PBS containing 50 mM Tris base, 150 mM NaCl, pH 7.4, and 0.05% Tween‐20). NeutrAvidin (10 µL of 0.2 mg mL^−1^ in PBST; Thermo Fisher, Cat. No. 31000) was added to each well and incubated for 10 min. Following this, a wash sequence was performed: two 10 µL washes with PBST, followed by one 10 µL wash with PBS containing 1% Tween‐20. Biotinylated capture antibodies were diluted to 10 nM in blocking solution (1 mg mL^−1^ BSA in PBS, filtered through 0.2 µm filter) and incubated in the wells for 10 min. After another wash sequence, wells were blocked with blocking solution for 10 min, followed by a single PBST wash. Samples were then added to the wells (10 µL per well) and incubated. Brain homogenates were diluted 1:10 in PBS and incubated at room temperature for 1 h. Primary neuron lysates were diluted 1:20 in PBS and incubated at room temperature for 1 h. Media samples were used neat and incubated overnight at 4 °C. This was followed by a wash sequence. Two differentially fluorescently labeled detection antibodies (anti‐tau antibody AF647 at 2 nM and anti‐interactor antibody AF488 at 2 nM) were combined in blocking solution and incubated for 15 min in the dark. A final wash sequence was performed, followed by a PBS wash. Wells were then filled with fresh PBS and sealed with a clean coverslip.

***SiMPull for DNA‐PAINT***: To adapt SiMPull to DNA‐PAINT, the protocol was carried out as described above, but instead of dye‐labeled detection antibody a DNA‐labeled detection antibody (DBCO TEG‐AAACCACCACCACCACCACCACCACCACCACCACCA, ATDBio) was used, and an additional blocking step was performed; after the sample incubation and a washing cycle, an additional 30 min blocking step with blocking solution was performed. DNA‐labelled detection antibody was then diluted in 0.1 mg mL^−1^ BSA in PBS (AT8: 5 nM, HT7: 2 nM, AL177: 5 nM) and incubated for 15 min. After a washing cycle and an additional PBS wash, 5 µL of imaging strand (1 nM, TGGTGGT‐Atto655, ATDBio) was added to each well and sealed with a clean coverslip.

### 5.9 Total Internal Reflection Fluorescence Microscopy

***Set‐up***: Fluorescent images were acquired using a custom‐built total internal reflection fluorescence (TIRF) microscope, as previously described [13]. The setup was based on a Nikon Ti‐2 Eclipse inverted microscope body, equipped with a 60×, 1.49 N.A. Apo TIRF objective and Nikon's Perfect Focus System. Excitation was provided by 638 nm (Cobolt 06‐MLD‐638), 561 nm (Cobolt 06‐DPL‐561), 488 nm (Cobolt 06‐MLD‐488), and 405 nm (Cobolt 06‐MLD‐405) lasers (all from HÜBNER). Detection antibodies conjugated to AF647 or AF488 were excited at 638 and 488 nm, respectively, DNA imaging strand conjugated to atto655 was excited at 638 nm. Emitted fluorescence was collected through the objective, passed through a quad‐band dichroic beam splitter, and filtered using the appropriate emission filters (for 488 nm: BLP01‐488R‐25 × 36 and FF01‐520/44‐25×36; for 638 nm: BLP01‐635R‐25 × 36; all from Semrock). Images were captured on an Evolve 512 EMCCD camera (Photometrics) operating in frame‐transfer mode, with an electron‐multiplying gain of 6.3 electrons/ADU and 250 ADU/photon. Each image was 512 × 512 pixels, where the pixel size corresponded to 107 nm in the recorded image. To minimize bias in field selection, images were acquired in a grid pattern using an automated script in Micro‐Manager.

For co‐localization imaging, the 638 nm laser was set to 5 mW and the 488 laser to 8 mW (1% – 2% of the maximum power). For each well, 16 fields of view were imaged each for 50 frames of 50 ms exposure. The images were acquired for each field of view sequentially in each excitation channel. For super‐resolved imaging, lasers were set to maximum power (190 mW 638 nm), with 4 fields of view imaged per well each for typically 8000 frames of 100 ms exposure.

### 5.10 Image Analysis

***Co‐localization image analysis***: For co‐localization experiments, images were acquired sequentially in each excitation channel in a 4 × 4 grid. Spot detection was performed independently in both channels using ComDet, yielding separate lists of centroid coordinates. Co‐localized spots were identified based on a Euclidean distance threshold of 4 pixels, assigning each spot in one channel to the nearest spot in the opposing channel if within this radius. Where multiple candidate matches were present, the closest was selected as the co‐localized pair. Chance co‐localization was estimated by transposing the centroid coordinates of the second channel along the x‐dimension and repeating the distance‐based analysis. The proportion of co‐localized spots was calculated as the number of matched spots divided by the total number of detected spots in the given channel.

***Super‐resolved image analysis***: Super‐resolution images were reconstructed using the Picasso package. After discarding the first 100 frames, localizations were identified, fitted, and corrected for microscope drift using redundant cross‐correlation. Localizations with a precision ≥30 nm were excluded. Aggregate clusters were obtained using DBSCAN implemented in scikit‐learn (radius = 0.3 (∼35 nm), minimum density = 5). Aggregate properties including area, perimeter, and eccentricity were determined with scikit‐image, while aggregate length was measured using SKAN, reporting the summed branch distance of skeletonized aggregates. Super‐resolved images were rendered with the built‐in Picasso functionality.

### 5.11 Proteomics Analysis

***Affinity precipitation and peptide preparation***: Affinity precipitation of aggregates from brain tissue homogenate samples was performed as previously described with small modifications [42]. Tau complexes were immunoprecipitated from five AD and five HC individuals (Table 1), but one AD sample was excluded from the proteomic analysis due to low yield. Briefly, depending on the antibody host species Protein A or Protein C conjugated Dynabeads (Dynabeads Protein A for rabbit, Dynabeads Protein C for mouse, both Invitrogen) were used. For each sample, 66.6 µL of beads were washed three times with 300 µL of PBS and then diluted to 100 µl in PBS.

For each sample, 16 µg of the respective antibodies (HT7, TOMA1, MC1, AT8, T181) was diluted in 100 µL PBS, and incubated with 100 µL of washed beads for 1 h at room temperature under rotation. After this, the supernatant was removed, and the beads were washed with PBST three times. The washed beads were resuspended in 100 µL of PBS. To confirm antibody binding to the beads, the supernatant and washes were tested using A280. For each brain homogenate sample, 20 µg of protein was diluted in 100 µL of PBS, mixed with 100 µl of the respective antibody‐beads, and incubated overnight at 4°C under rotation. After incubation, the beads were separated from the depleted supernatant using a magnet and the supernatant was kept. Beads were washed twice with PBS to minimize non‐specific background.

For the total samples, total protein was precipitated from brain homogenate by combining 100 µL of sample with 5× ice‐cold acetone and incubating overnight at −20°C. The precipitated proteins were then isolated by centrifugation (20 000 × g for 30 min at 4°C).

The total protein, the affinity‐captured aggregates and the depleted supernatant samples were then prepared for mass spectrometry analysis, alongside pooled reference samples generated by combining equal amounts of peptide material from all samples. This reference sample was included in each TMT set. For this, the iST‐NHS (Preomics) and tandem mass tag 11‐plex (ThermoFisher Scientific) kits were used according to the manufacturer's instructions. Briefly, samples were lysed and denatured at 95°C, followed by tryptic digestion and TMT labelling. Affinity‐captured proteins were processed directly on‐bead; bead‐bound proteins were lysed and subjected to on‐bead tryptic digestion, after which beads were removed and peptides collected for downstream analysis. The peptides were quantified via BCA assay (ThermoFisher Scientific) and the final concentration of peptides was adjusted to 0.1 µg µl^−1^ in LC‐LOAD buffer (Preomics).

***Mass spectrometry analysis***: Prior to mass spectrometry analysis, the peptide fractions were dried down and reconstituted in 0.1% formic acid (Thermo Fisher). Samples were analyzed using an Orbitrap Fusion Lumos Tribrid mass spectrometer coupled to an UltiMate 3000 RSLCnano liquid chromatography system. Approximately 1 µg of peptide material was loaded onto a 2 cm PepMap C18 100Å trapping column (300 µm internal diameter, Thermo Fisher) at a flow rate of 5 µl min^−1^ using the loading pump. Peptides were subsequently separated on a 50 cm EASY‐Spray analytical column (75 µm internal diameter, Thermo Fisher) operated at a flow rate of 300 nL min^−1^. The analytical column was connected to the mass spectrometer via an EASY‐Spray nanoelectrospray ion source. Chromatographic separation was achieved using a linear gradient of solvent A (0.1% formic acid in water) and solvent B (0.1% formic acid in 99.9% acetonitrile, Thermo Fisher). The gradient consisted of an increase from 3% to 30% solvent B over 105 min, followed by 30% to 40% over 15 min, then 40% to 99% over 5 min. The column was held at 90% solvent B for 10 min before being re‐equilibrated at 3% solvent B for an additional 10 min, yielding a total run time of 140 min, including a 120 min analytical gradient. Data were acquired in data‐dependent acquisition (DDA) mode. For quantification, synchronous precursor selection (SPS)‐based MS^3^ fragmentation was employed to capture TMT reporter ions.

***Peptide identification and further analysis***: Peptide identification and quantification were performed using MaxQuant (version 2.6.2.0), searching against the SwissProt *Homo sapiens* database (downloaded 27 February 2024; 20418 entries). The search parameters included a mass tolerance of 20 ppm for MS and 0.6 Da for MS/MS, permitting one missed cleavage and enabling the “match between runs” feature. Methionine oxidation and N‐terminal protein acetylation were specified as variable modifications, while cysteine alkylation by PreOmics reagents (+113.084 Da) was defined as a fixed modification. The false discovery rate was set to a maximum of 0.005 (0.5%) at the peptide level and 1% at the protein level. All remaining settings were kept at their default values.

Further downstream analysis was carried out using custom Python scripts [42, 43]. Common contaminants, including keratins, were excluded. Proteins were retained for quantification only if they were identified by at least two peptides, with a minimum of one being unique. Protein abundances were normalized either per run (for total protein analysis) or per TMT channel (for affinity‐enriched aggregates) relative to the pooled reference channel included in each TMT set, based on reporter ion intensities. For each AD sample, abundance ratios were computed relative to the corresponding mean abundance in control samples. In the case of affinity‐captured proteins, these ratios were additionally normalized to the total protein ratio to correct for loading differences. The resulting abundance ratios were tested using a one‐sample t‐test against a null hypothesis mean of 1, representing no change between disease and control. To identify proteins with differential abundance in AD, we applied thresholds of |log_2_(abundance)| > 1 and –log_10_(*p*‐value) > 1.3. Protein‐level p values were not adjusted for multiple testing; these thresholds were used for exploratory candidate prioritization rather than confirmatory statistical inference. GO term enrichment analysis was performed using a custom Python function that applied Fisher's exact test with Bonferroni correction to identify significantly enriched biological processes, molecular functions, and cellular components for each protein set across columns, based on the PANTHER GO‐Slim ontology. A STRING search was performed using Cytoscape (string version: 2.0.3) with a confidence level of 0.4.

## Author Contributions

Conceptualization, **D.B**. and **D.K**.; methodology, **D.B**. and **D.C**.; investigation, **D.B**., **P.B.L**., **D.C**., **Y.W**., **E.F**.; writing – original draft, **D.B**.; writing – review & editing, all authors; funding acquisition, **D.B**. and **D.K**.; resources, **L.A.R**., **C.L.C**.; supervision, **D.K**.

## Conflicts of Interest

The authors declare no conflicts of interest.

## Supporting information




**Supporting File**: advs77822‐sup‐0001‐SuppMat.pdf.

## Data Availability

All original code has been deposited at Zenodo at https://doi.org/10.5281/zenodo.17990783 and is publicly available as of the date of publication. The mass spectrometry proteomics data have been deposited to the ProteomeXchange Consortium via the PRIDE [37] partner repository with the dataset identifier PXD072117. The PRIDE dataset includes the raw mass‐spectrometry files, the MaxQuant parameter file, and the corresponding MaxQuant output files. All data are available in the manuscript or the supplementary material. Raw data available on written request to DK.
