# Supplementary material for: Temporal Trajectories of the Tau Aggregate Interactome Reveal Stage‐Specific Vulnerabilities in Alzheimer's Disease
Source: Adv Sci (Weinh). 2026 Sep 16:e77822. Online ahead of print. doi: 10.1002/advs.77822 (PMC13583081; doi:10.1002/advs.77822)
Supplement: Supplementary file 1 [file ADVS-9999-e77822-s001.pdf]

Supplemental data for “Temporal Trajectories of the Tau Aggregate Interactome Reveal Stage-Specific Vulnerabilities in Alzheimer’s Disease”

Böken *et al.*

Figure S1 HT7, TOMA1 and MC1 validation

Figure S2 Patient interactor consistency

Figure S3 Proteomics total brain homogenate and supernatant

Figure S4 Simoa and LDH, BCA, ASC

Figure S5 pT217 and pT181 profiles of TOMA1 and MC1 tau

Figure S6 Co-localisation data neurons

Figure S7 Tau-MAP2 co-localisation neurons

Figure S8 Brightness validation

Figure S9 Proteinase K digestion

Figure S10 Co-localisation data in brain homogenate

Figure S11 P-tau pseudotime analysis

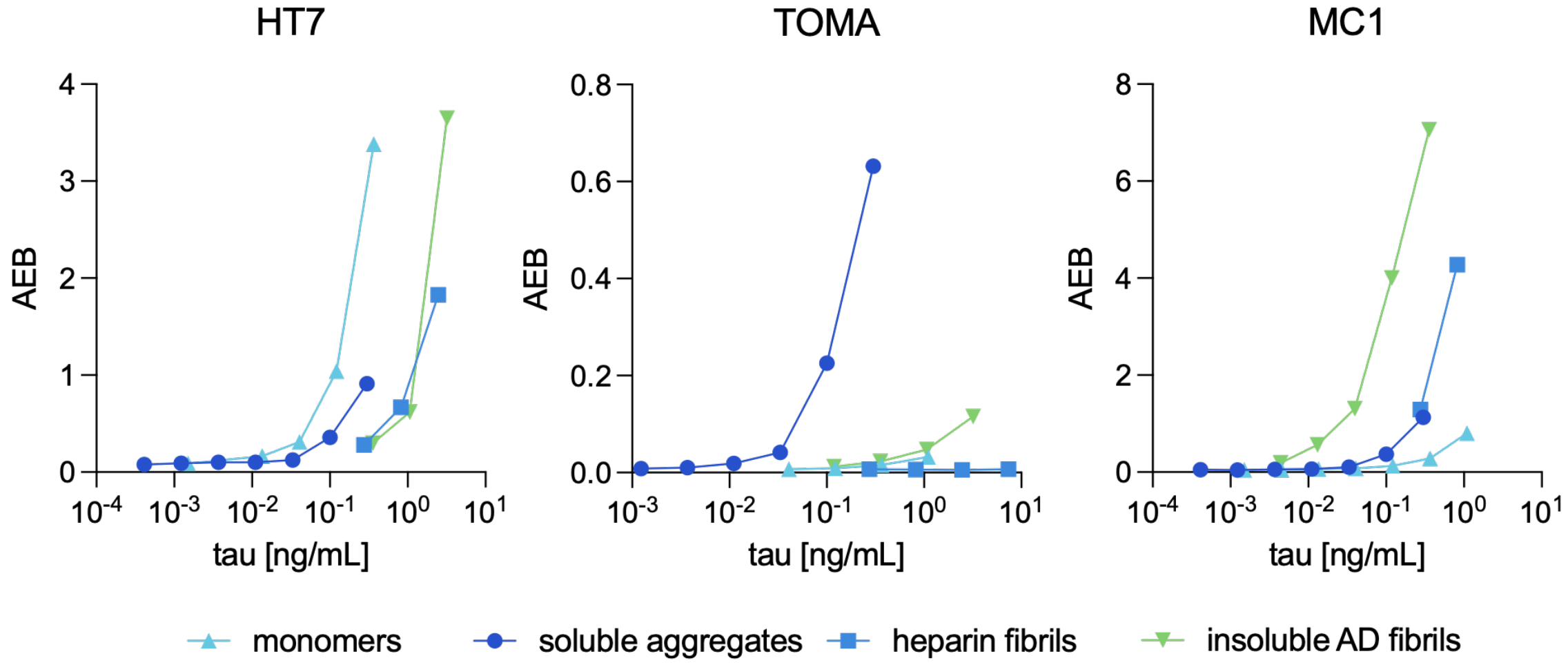

Supplemental Figure S1: Orthogonal characterisation of HT7, TOMA1 and MC1 reactivity across tau assembly states using Simoa. Monomeric tau, soluble hyperphosphorylated tau aggregates, heparin-assembled tau fibrils and sarkosyl-insoluble tau aggregates isolated from Alzheimer's disease brain tissue were analysed using Simoa immunoassays with HT7, TOMA1 or MC1 as the capture antibody and the pan-tau antibody Tau5 for detection.

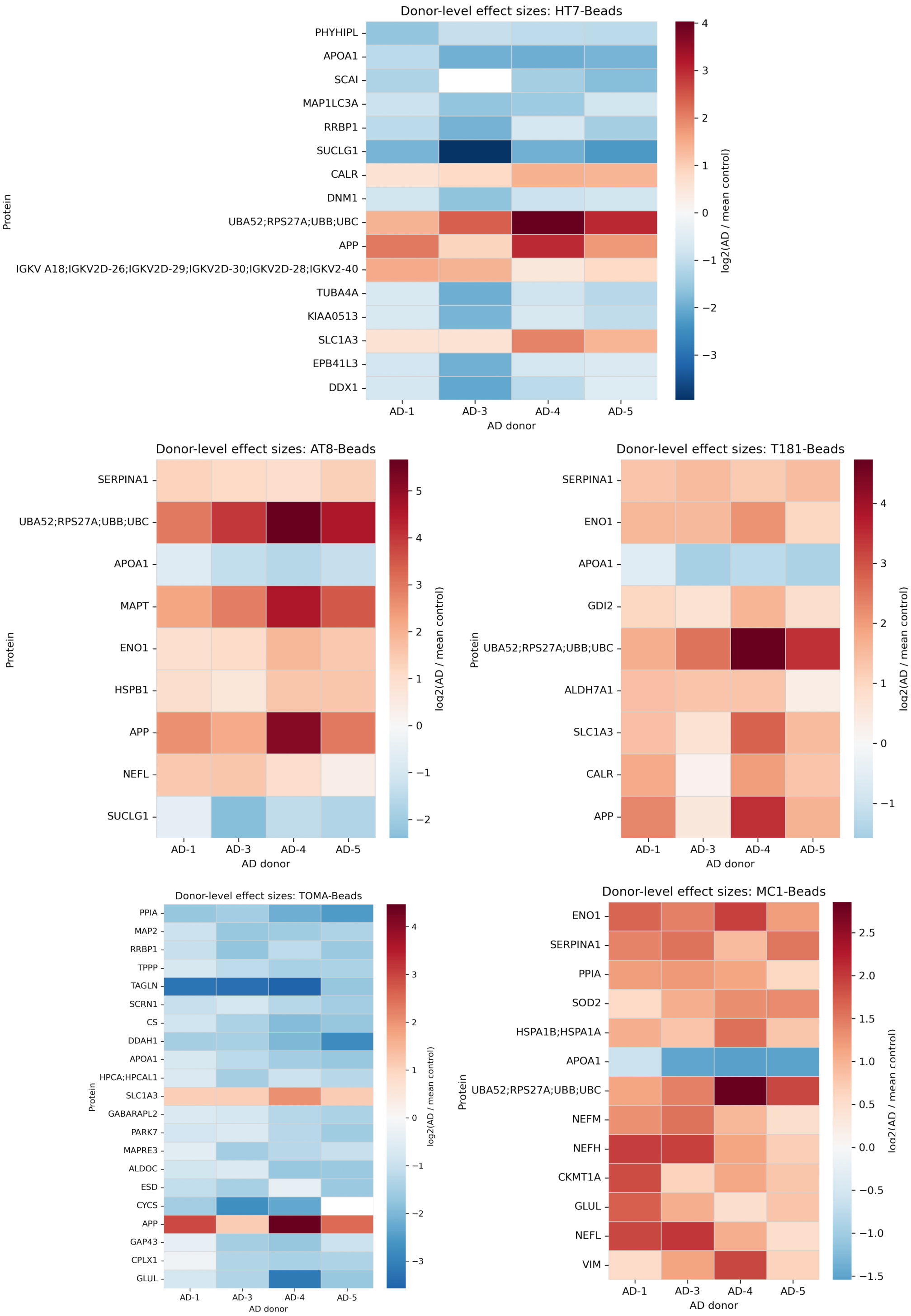

Supplemental Figure S2: Donor-level consistency of altered interactors in Alzheimer's disease. Heatmap showing the donor-level log2 abundance ratio relative to the mean control abundance for the significant proteins identified in the pulldowns using HT7, AT8, T181, TOMA1, MC1. Proteins were ranked by nominal p value and effect size. Red and blue indicate increased/decreased abundance in AD, respectively. The broadly concordant changes across donors indicate that the observed effects were not driven by a single individual.

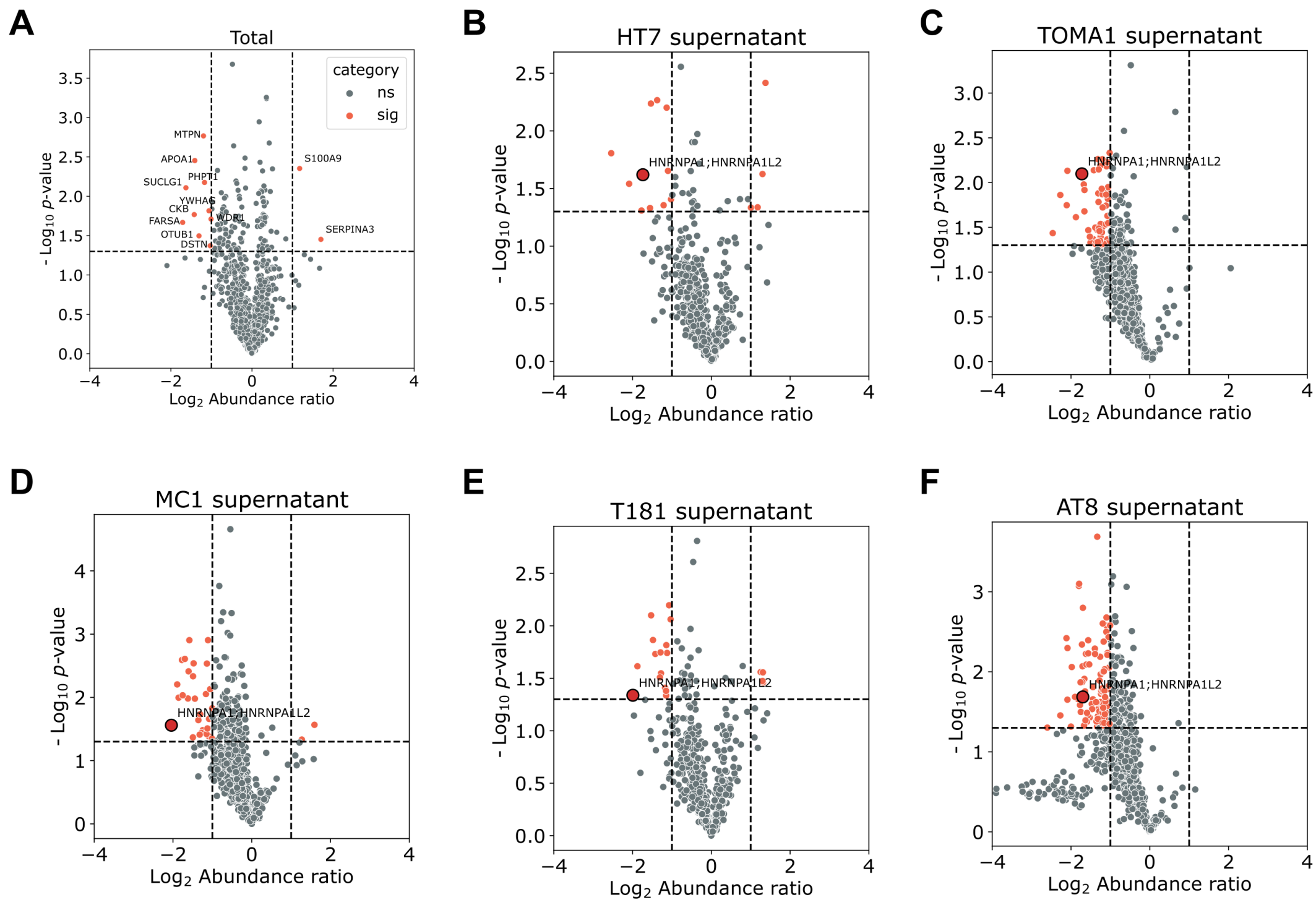

Supplemental Figure S3: Volcano plots of proteomics experiments on total brain homogenate and depleted supernatants. Log2 abundance ratio between AD (n = 5) and healthy control brain (n = 5) homogenate samples from (A) total brain homogenates and (B-F) depleted supernatants after immunoprecipitation with (B) HT7 antibody, (C) TOMA1 antibody, (D) MC1 antibody, (E) T181 antibody and (F) AT8 supernatant.

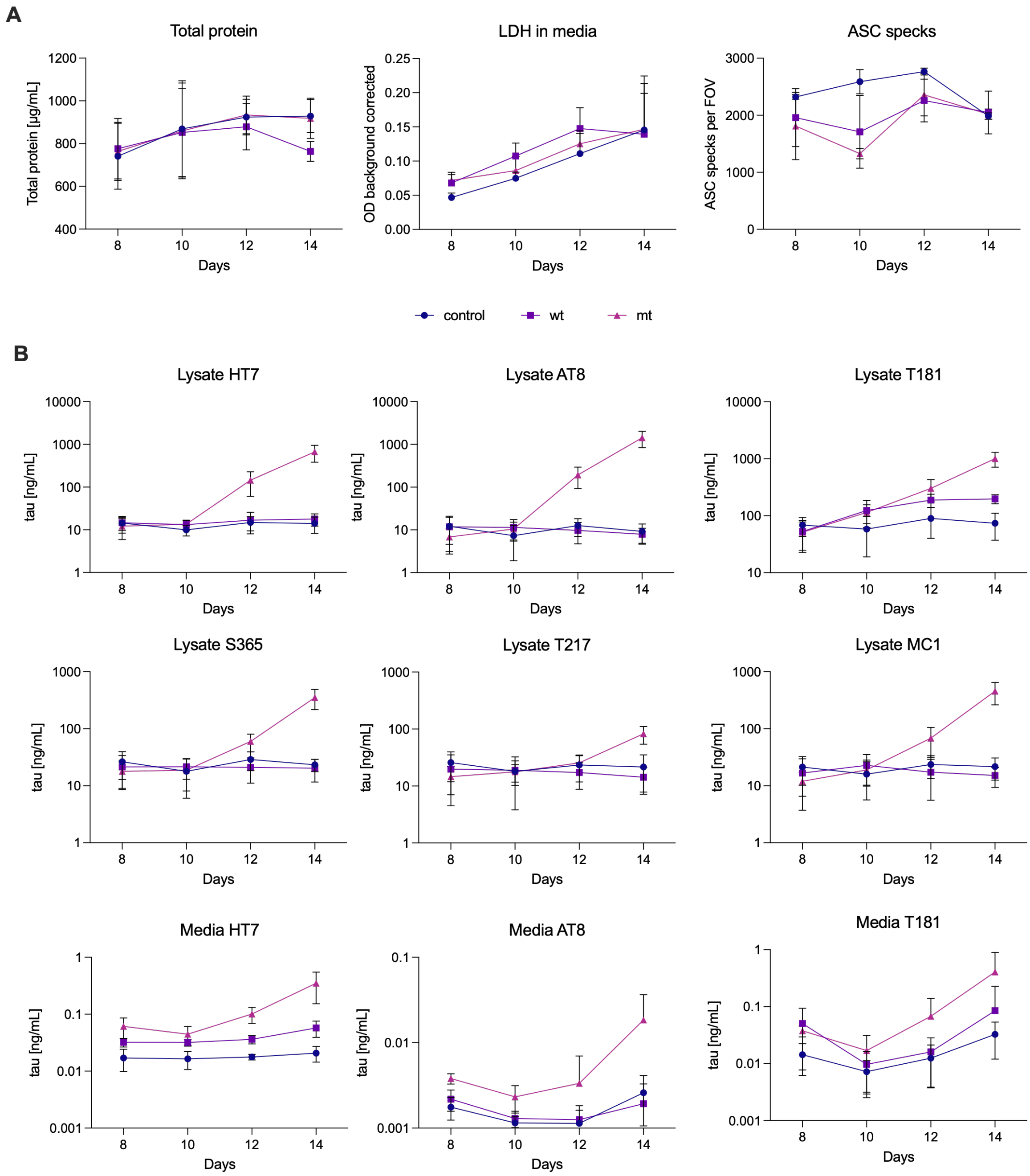

Supplemental Figure S4: Characterisation of primary mouse neurons expressing human wt or P301S/S320F tau. (A) Markers of general cell health were measured, including levels of total protein in the cell lysate, LDH in the media and ASC specks in the lysate of cells transduced with either wildtype tau (wt), P301/S320F tau (mt) or non-transduced controls (control). (B) Tau aggregate levels determined by Simoa using various tau and p-tau specific antibodies. Values are shown as the mean ± S.D. of three biological replicates, each consisting of two technical replicates.

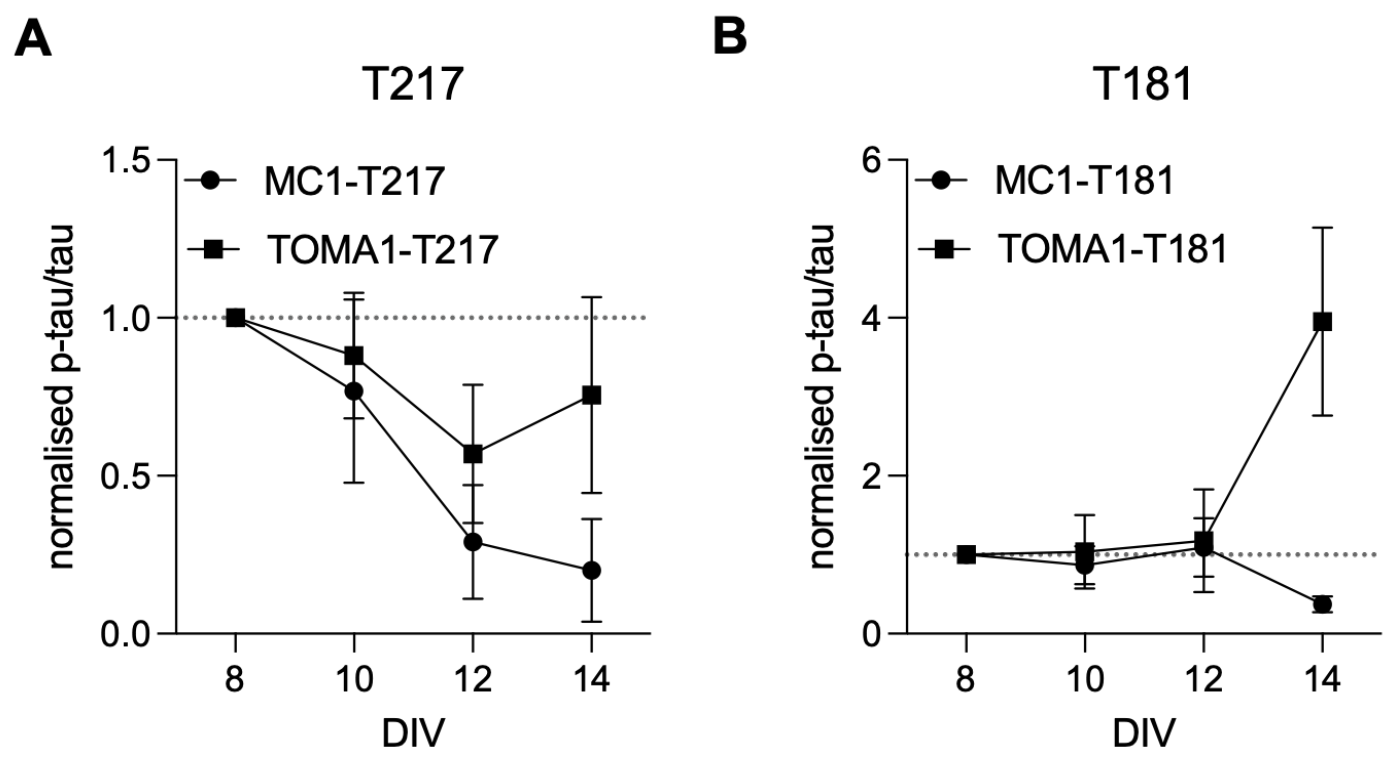

Supplemental Figure S5: Temporal pT181 and pT217 profiles in TOMA1- and MC1-captured tau species. P301L/S320F tau-expressing neuronal lysates were analysed by Simoa using TOMA1 or MC1 capture and (A) pT217 or (B) pT181 detection. Signals were first normalised to the corresponding levels of TOMA1- or MC1-positive tau species, and then to day 1 of the time course. Data are shown as mean ± SD from n = 3 biological replicates.

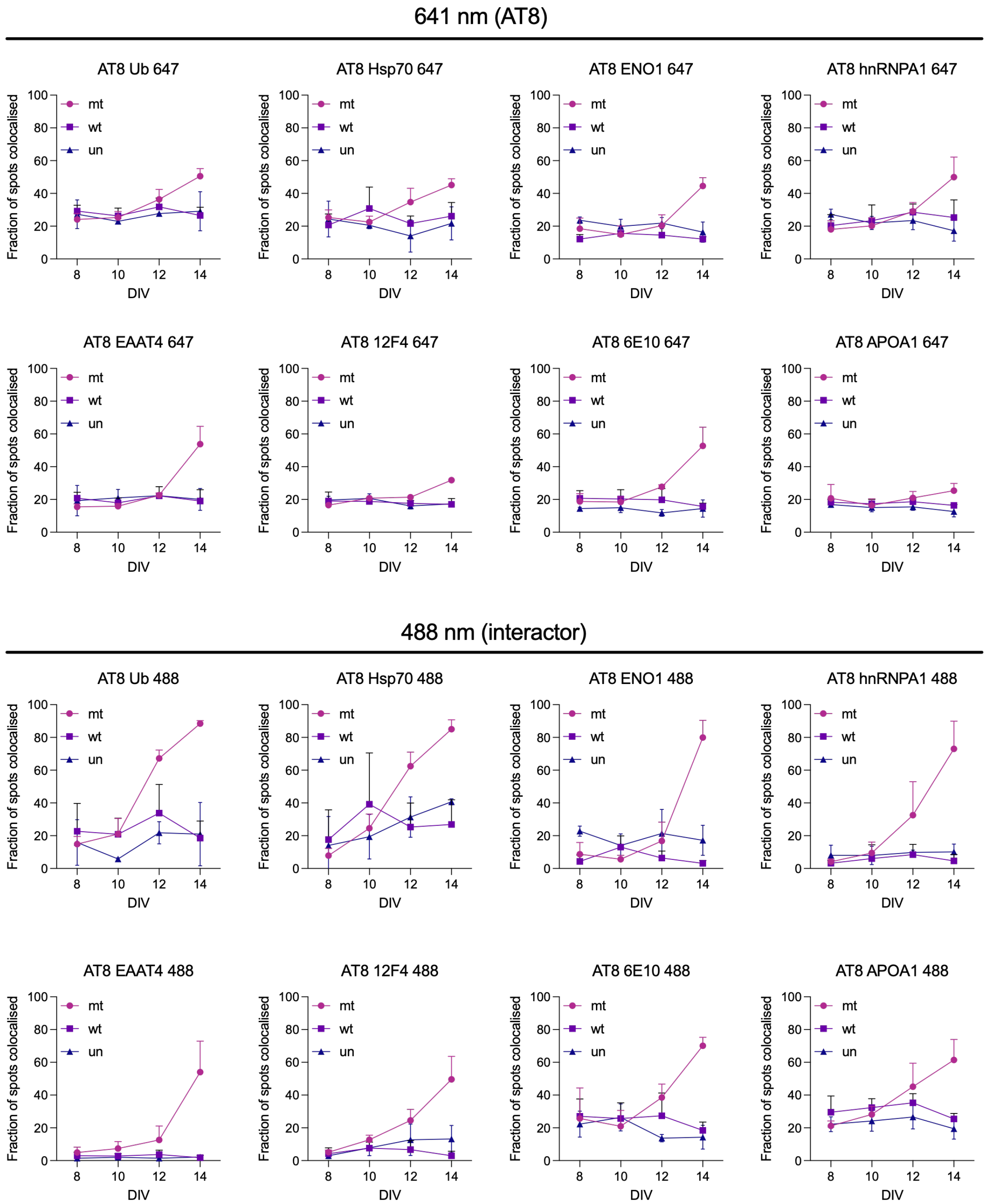

Supplemental Figure S6: Co-localisation analysis of tau aggregates with key interactors in mouse primary neurons expressing human tau. AT8 antibody was used for capture and detection (638 channel) as well as 488-labelled antibody for the respective interactors. Mt: human mutant, wt: human wt tau, un: untransfected control. Values are shown as mean ± S.D. of three biological replicates, each consisting of two technical replicates.

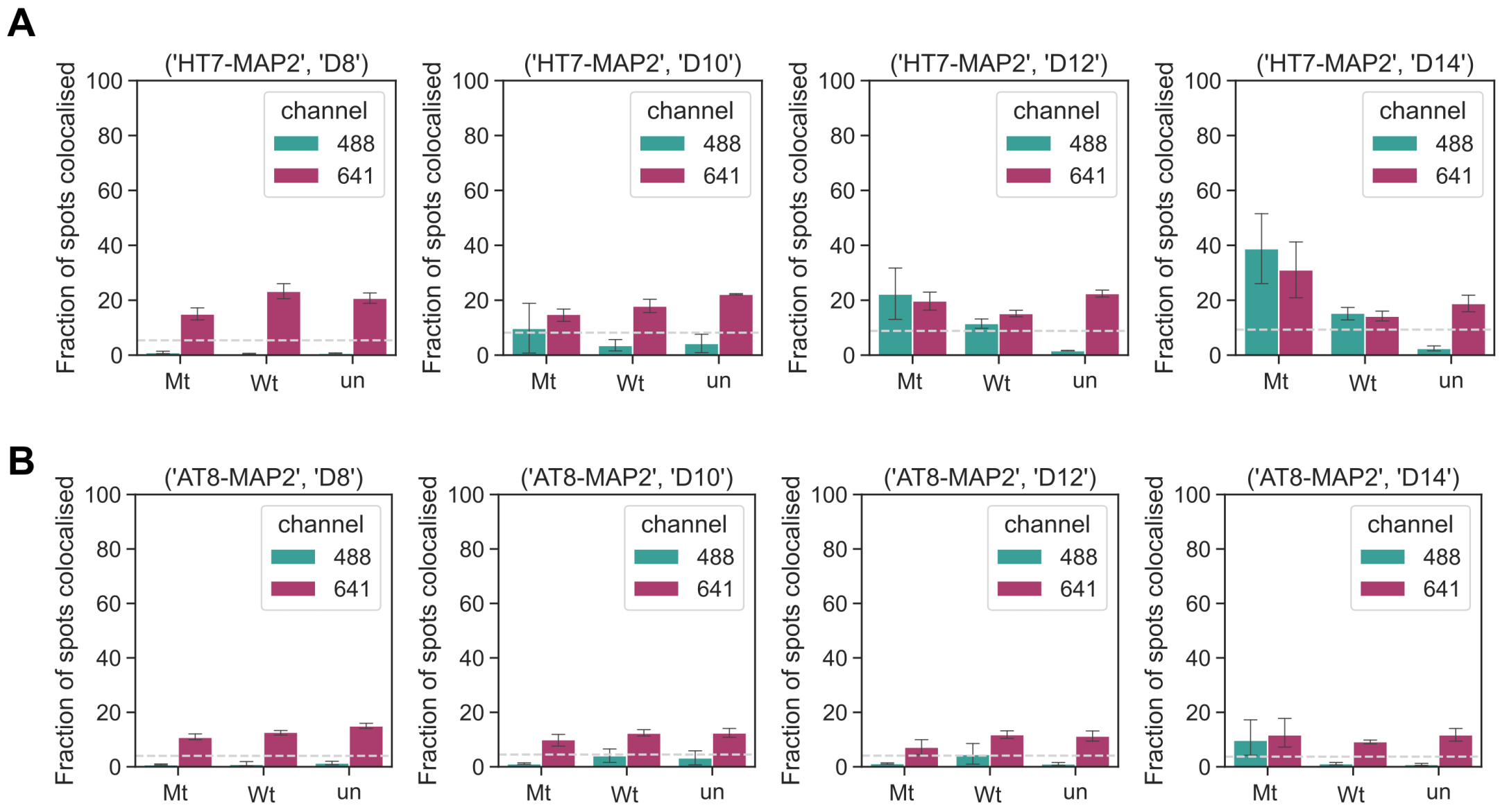

Supplemental Figure S7: Co-localisation analysis of tau aggregates with MAP2 interactors in mouse primary neurons expressing human tau. Matching tau antibodies were used for capture and detection (638 channel) as well as 488-labelled antibody for the respective interactors; (A) HT7 capture, (B) AT8 capture. Values are shown as mean ± S.D. of two biological replicates, each consisting of two technical replicates.

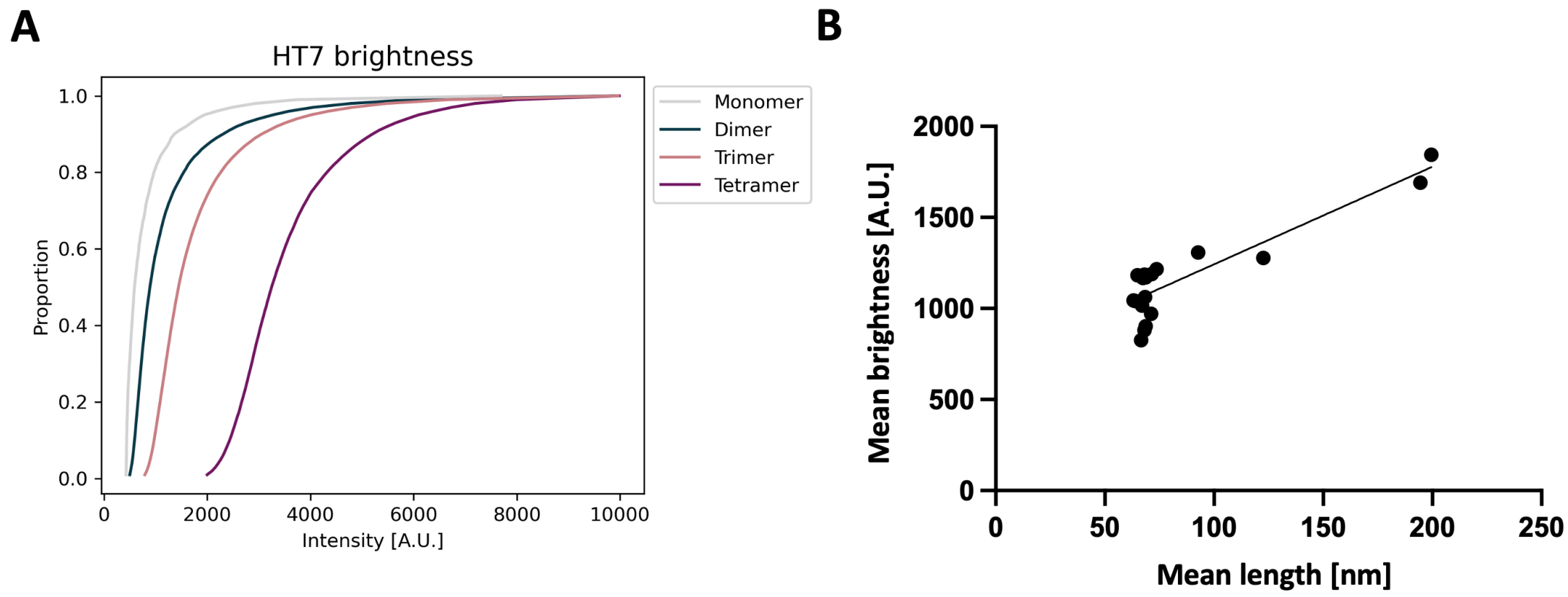

Supplemental Figure S8: Validation of fluorescence brightness as a relative proxy for tau aggregate size. (A) Cumulative distribution functions of fluorescence intensity measured by HT7 SiMPull for defined synthetic tau constructs containing increasing numbers of accessible tau epitopes, from monomer to tetramer. (B) Relationship between mean SiMPull fluorescence brightness and mean aggregate length measured independently by DNA-PAINT across matched samples (Mt tau cell lines).

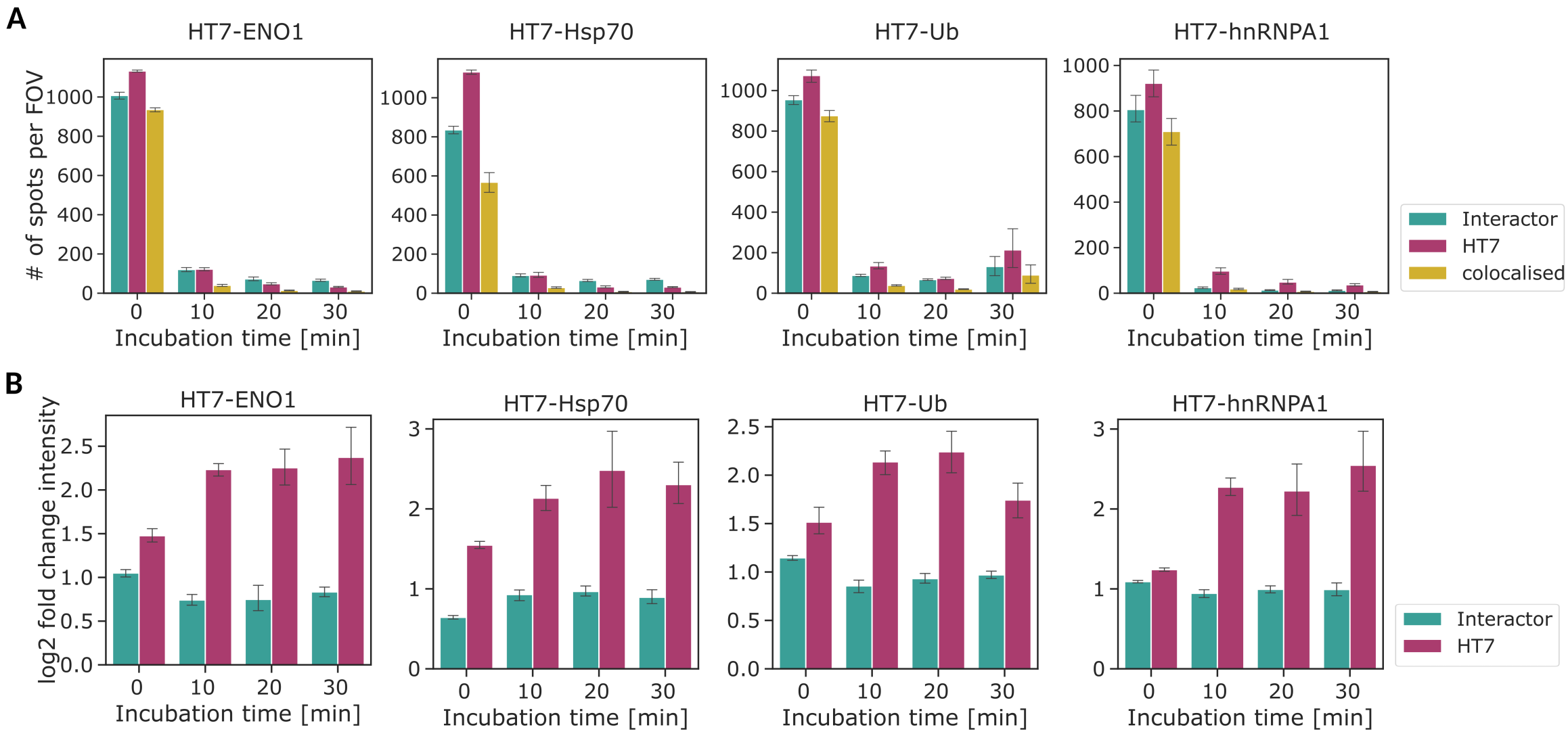

Supplemental Figure S9: Co-localisation analysis of tau aggregates with key interactors after Proteinase K treatment. Pooled (n=5) AD brain homogenate samples were incubated with Proteinase K for 0-30 min before quenching with PMSF. Matching tau antibody (HT7) were used for capture and detection (638 channel) as well as 488-labelled antibody for the respective interactors. (A) Number of spots in each field of view (FOV) for the interactor, the tau aggregates and the co-localised spots. (B) Log2 fold change of the brightness of co-localised vs non-colocalised spots. Values are shown as mean ± S.D. of 16 images per condition.

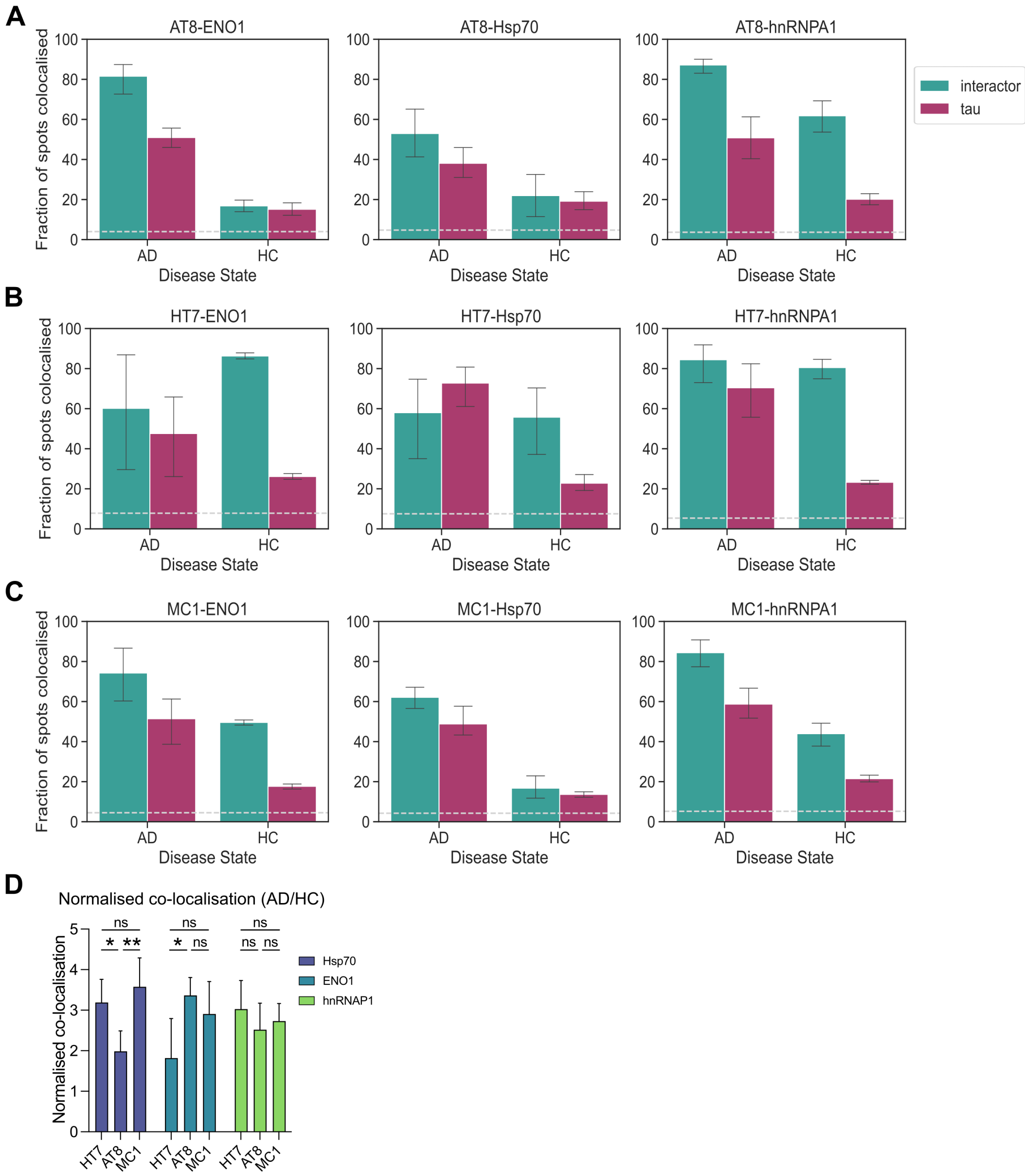

Supplemental Figure S10: Co-localisation analysis of tau aggregates with key interactors in human AD and healthy control samples. Matching tau antibodies were used for capture and detection (638 channel) as well as 488-labelled antibody for the respective interactors; (A) AT8 capture, (B) HT7 capture, (C) MC1 capture. Values are shown as mean ± S.D. of five biological replicates per disease group. (D) Normalised co-localisation (AD/HC) of HT7, TOMA1, and MC1 with ENO1, Hsp70, and hnRNPA1. Bars show mean ± SD. p values from two-way ANOVA with Tukey post hoc tests. (C) Heatmap with hierarchical clustering based on fold changes in interacting proteins between aggregation states, revealing four clusters with shared tau-interaction dynamics (n = 4 AD, n = 5 HC).

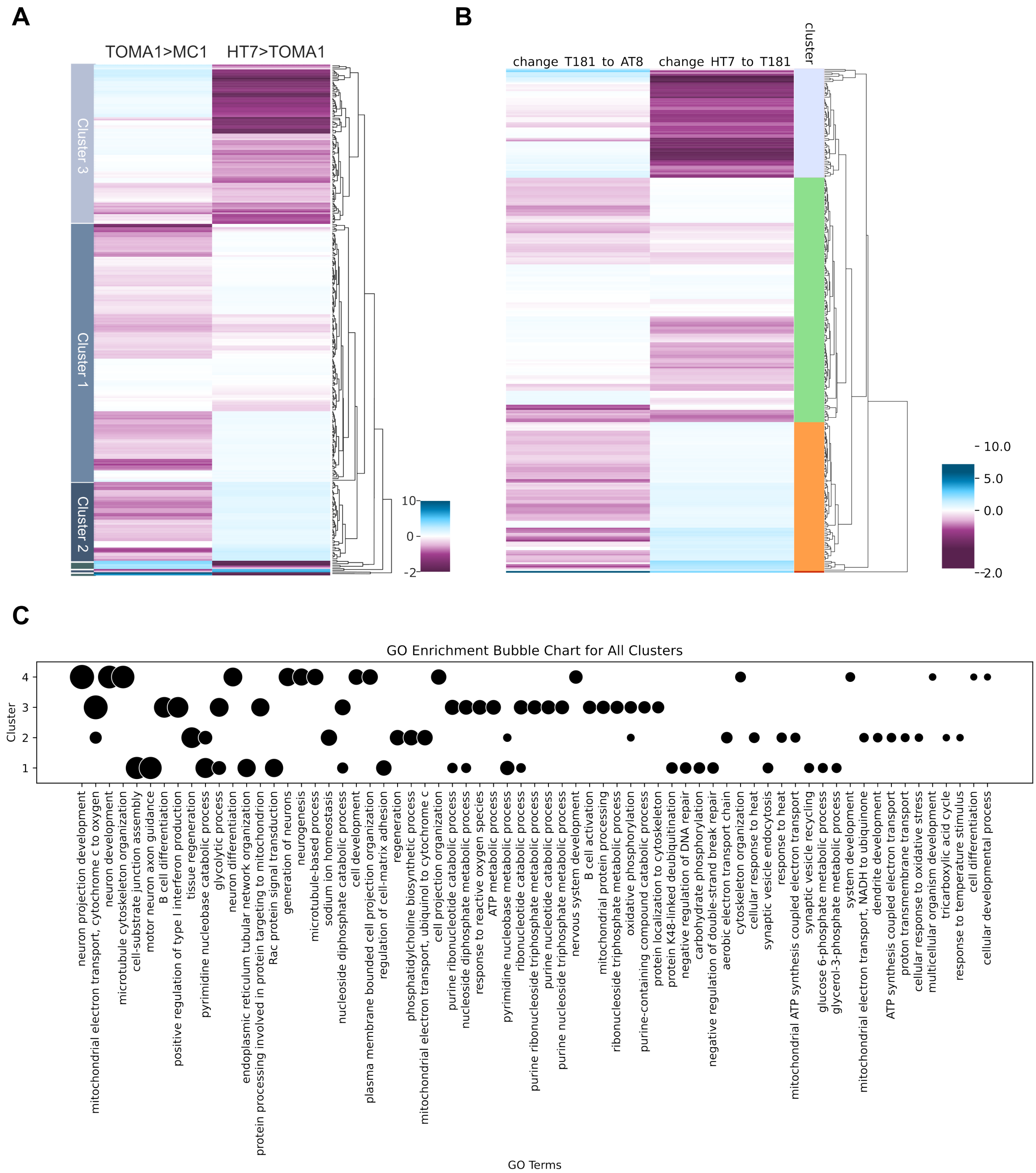

Supplemental Figure S11: Pseudotime analysis of aggregation and p-tau specific interactomes. Progression of tau-protein interactions from HT7 – TOMA1 – MC1 and HT7 – T181 – AT8. (A) Heatmap with hierarchical clustering based on fold changes in interacting proteins between aggregation states, revealing four clusters with shared tau-interaction dynamics (n = 4 AD, n = 5 HC). (B) Heat map clustering for the identification of protein groups with similar changes in association with tau with phosphorylation. Cluster 1: orange, cluster 2: green, cluster 3: blue, cluster 4: red. (C) GO enrichment analysis for protein clusters with similar interaction patterns across phosphorylation stages.
